# MiR-29b-3p Inhibits Migration and Invasion of Papillary Thyroid Carcinoma by Downregulating COL1A1 and COL5A1

**DOI:** 10.3389/fonc.2022.837581

**Published:** 2022-04-22

**Authors:** Congjun Wang, Ye Wang, Zhao Fu, Weijia Huang, Zhu Yu, Jiancheng Wang, Kaitian Zheng, Siwen Zhang, Shen Li, Junqiang Chen

**Affiliations:** ^1^ Department of Gastrointestinal Gland Surgery, The First Affiliated Hospital of Guangxi Medical University, Nanning, China; ^2^ Clinical Research Lab, Guangxi Key Laboratory of Enhanced Recovery After Surgery for Gastrointestinal Cancer, Nanning, China; ^3^ Department of Gastrointestinal, Hernia and Enterofistula Surgery, The People’s Hospital of Guangxi Zhuang Autonomous Region, Nanning, China

**Keywords:** microRNAs, miR-29b-3p, papillary thyroid carcinoma, downregulation, COL1A1, COL5A1, gene expression

## Abstract

**Introduction:**

MicroRNAs (miRNAs) are small noncoding RNA molecules that regulate genetic expression and are also vital for tumor initiation and development. MiR-29b-3p was found to be involved in regulating various biological processes of tumors, including tumor cell proliferation, metastasis, and apoptosis inhibition; however, the biofunction and molecule-level mechanisms of miR-29b-3p inpapillary thyroid carcinoma (PTC) remain unclear.

**Methods:**

The expression of miR-29b-3p in PTC samples was tested *via* qRT-PCR. Cellular proliferation was analyzed by CCK-8 and EdU assays, and cellular migratory and invasive abilities were assessed utilizing wound-healing and Transwell assays. In addition, protein expressions of COL1A1, COL5A1, E-cadherin, N-cadherin, Snail, and Vimentin were identified *via* Western blot (WB) assay. Bioinformatics, qRT-PCR, WB, and dual luciferase reporter assays were completed to identify whether miR-29b-3p targeted COL1A1 and COL5A1. In addition, our team explored the treatment effects of miR-29b-3p on a murine heterograft model.

**Results:**

Our findings revealed that miR-29b-3p proved much more regulated downward in PTC tissue specimens than in adjacent non-cancerous tissues. Meanwhile, decreased expression of miR-29b-3p was strongly related to the TNM stage of PTC patients (p<0.001), while overexpression of miR-29b-3p in PTC cells suppressed cellular migration, invasion, proliferation, and EMT. Conversely, silencing miR-29b-3p yielded the opposite effect. COL1A1 and COL5A1 were affirmed as the target of miR-29b-3p. Additionally, the COL1A1 and COL5A1 were highly expressed in PTC tumor samples than in contrast to neighboring healthy samples. Functional assays revealed that overexpression of COL1A1 or COL5A1 reversed the suppressive role of miR-29b-3p in migration, invasion, and EMT of PTC cells. Finally, miR-29b-3p agomir treatment dramatically inhibited Xenograft tumor growth in the animal model.

**Conclusions:**

These findings document that miR-29b-3p inhibited PTC cells invasion and metastasis by targeting COL1A1 and COL5A1; this study also sparks new ideas for risk assessment and miRNA replacement therapy in PTC.

## Introduction

Papillary thyroid carcinoma (PTC) is the most common primary malignancy of the endocrine system, with the incidence increasing year by year worldwide (1, 2). Its prevalence among female individuals across the world is 10.2 per 100,000 individuals, which constitutes threefold that of male individuals; however, PTC patients demonstrate excellent long-term prognosis (3, 4). About 30%–65% of PTC cases feature performed lymph node metastasis, and <10% of PTC cases have shown distant metastasis in the bones and lungs (5). Although surgical resection remains the most effective treatment for PTC, and active surveillance is suggested for select low-risk PTC patients, according to the 2015 ATA guidelines, a reluctance to offer it continues due to lack of robust evidence or protocols (6). On the other hand, the clinical outcomes of PTC patients attended by high invasion and proliferation remain poor (7). Approximately 5%–10% of PTC sufferers encounter relapse in 5 years of primary treatment, which reduces patient survival (8). For that reason, it remains vital to expose the potential causal link between PTC development and etiopathogenesis, which might deliver us additional PTC regimens.

MiRNAs are endogenetic single-stranded non-coding RNA molecules that bind to protein-coding genes by means of 3′-untranslated region (3′-UTR) of target messenger RNA mediating post-transcriptional gene expression (9, 10). An ever-growing number of proofs demonstrate that miRNAs participate in the biological processes of cancer (11, 12). Studies have revealed that miR-29b-3p is involved in the initiation and progress of various cancers, including pulmonary carcinoma, prostate cancer, cholangiocarcinoma, pancreatic cancer, and bladder cancer (13–17). Dysregulation of miR-29b-3p expression has been implicated in cell promotion, migration, and invasion in these tumors. Nevertheless, the effects of miR-29b-3p on thyroid carcinoma remain elusive.

Extra-cellular matrix (ECM) is closely correlated with tumor progression (18). Collagens, the main protein component of ECM, exhibit a variety of effects, demonstrating proliferative, migratory, and differentiative cellular activities *in vivo* (19). In turn, miR-29b-3p is a major regulator of the collagen family, which has been studied in a plurality of basic studies in the context of a range of diseases (20–22). Nevertheless, no studies regarding the regulation of collagen by miR-29b-3p in PTC are found in the literature.

The present research demonstrated that miR-29b-3p was low expressed in PTC samples, which was negatively associated with the proliferative and metastatic abilities of PTC cells. Furthermore, we confirmed that COL1A1 and COL5A1 are two immediate targets of miR-29b-3p and participated in the effects of miR-29b-3p on the biological capacity of PTC cells.

Finally, we contended that miR-29b-3p may serve as an underlying target for PTC treatment and exert pivotal effects on progression.

## Materials and Methods

### Bioinformatics Analysis

MicroRNA expression data retrieved from the Gene Expression Omnibus (GEO) database were collected to compare miR-29b-3p expression between human PTC samples and their adjacent normal, benign thyroid tissues. The GEO database (GSE15740) was adopted in order to analyze the expression of genes. Subsequently, differential analysis was carried out on differentially expressed genes with R language in the “limma” R package of Bioconductor, by screening criteria, *viz.*, |logFC| > 1.8 and p-value < 0.05. Furthermore, heatmaps were generated in R, with the “ggplots” R package.

mRNA expression (mRNA SeqV2) data of human PTC were extracted from The Cancer Genome Atlas (TCGA) database. Furthermore, we predicted the downstream target gene of miR-29b-3p through the online prediction software miRmap, microTissue, miRanda, PicTar, and TargetScan to determine the downstream target gene of miR-29b-3p. Thereafter, a protein–protein interaction (PPI) net was established *via* the STRING (https://string-db.org/) database. Gene Ontology (GO) and Kyoto Encyclopedia of Genes and Genomes (KEGG) assays were conducted employing the GO database (http://geneontology.org) and the KEGG database (http://www.genome.jp/kegg/), respectively.

### Patients and Tissue Samples

From January 2020 to November 2020, fresh tissue samples (PTC tumor and neighboring healthy samples) were harvested from PTC sufferers from The First Affiliated Hospital of GuangXi Medical University, none of whom had received any adjuvant treatment before surgery, with respect to which, it bears noting that the present research was accepted by the Ethical Board of The First Affiliated Hospital of GuangXi Medical University (Nanning, China). A total of 48 pairs of tissue samples were collected; the specimens were maintained in liquid nitrogen for long-term preservation and stored at −80°C for short-term assays.

### Cell Culture and Transfection

Human PTC (K1, TPC-1, KTC-1, and B-CPAP) and immortalized thyroid lineage cells (Nthyori3-1) purchased from the Type Culture Collection of the Chinese Academy of Sciences (Shanghai, China) were cultivated in Roswell Park Memorial Institute (RPMI) intermediary 1640 basic (RPMI 1640) (Thermo Fisher Scientific, MA, USA) and DMEM (Gibco, CA, USA) mixed with 100 mg/L streptomycins, 100 kU/L penicillin, and 10% fetal bovine serum (FBS) (HyClone, Logan, UT, USA) and kept in a 37°C incubator at 5% carbon dioxide.

The mimics/inhibitors of miR-29b-3p (miR-29b-3p mimic/miR-29b-3p inhibitor) and negative control (NC mimic/NC inhibitor) were purchased from Ribobio (RiboBio, Guangzhou, China). The small interfering RNAs of COL1A1 (si-COL1A1), COL5A1 (si-COL5A1), and negative control (NC) (GenePharma, Shanghai, China) were adopted to transfect the PTC cells with Lipofectamine 8000 reagent (Invitrogen, CA, USA). Overexpression of COL5A1 and COL1A1 was achieved by transfecting pcDNA 3.1 vector-cloned with gene sequences of COL5A1 and COL1A1 into PTC cells with Lipofectamine 3000 reagent (Thermo Fisher Scientific, MA, USA). Subsequently, these transfected PTC cells were cultivated for 24–48h before all the experiments herein were conducted.

### qRT-PCR

This study adopted a Fastpure Cell/Tissue Overall RNA Separation tool V2 (Vazyme, Nanjing, China) to extract all the RNAs and a reverse transcriptase tool (Vazyme, Nanjing, China) to prepare the cDNAs of COL5A1 and COL1A1 and miR-29b-3p; qRT-PCR was completed *via* the real-time fluorescent quantitation PCR equipment (ABI7500, America) with the SYBR qPCR Master Mix tool kit (Vazyme, Nanjing, China). The relative miR-29b-3p, COL1A1, and COL5A1 were identified *via* applying the 2^−ΔΔCt^ formula, where U6 and glyceraldehyde 3-phosphate dehydrogenase (GAPDH) were regarded as the internal references. The sequences of the entire primers herein are shown in **Table 1**.

**Table 1:** The sequences of the primers used for this study.

Genes	Primer sequences (5′–3′)
miR-29b-3p	(Sense) GCGGCGGTAGCACCATTTGAAATC
	(Antisense) GTCGTATCCAGTGCAGGGT
COL1A1	(Sense) GAGGGCCAAGACGAAGACATC
	(Antisense) CAGATCACGTCATCGCACAAC
COL5A1	(Sense) GCCCGGATGTCGCTTACAG
	(Antisense) AAATGCAGACGCAGGGTACAG
GAPDH	(Sense) ATTCCATGGCACCGTCAAGGCTGA
	(Antisense) TTCTCCATGGTGGTGAAGACGCCA
U6	(Sense) CGCTTCGGCAGCACATATAC
	(Antisense) TTCACGAATTTGCGTGTCAT

### Western Blot

Initially, a radio-immunoprecipitation assay (RIPA) lysis buffering solution (Beyotime, Shanghai, China) was applied to abstract proteins. Following the separation of proteins by sodium salt-polyacrylamide gel electrophoresis, target proteins were electrophoretically moved to nitrocellulose films, blocked *via* 5% milk-TBST for 2 h under room temperature. Subsequently, they were washed three times in TBST buffering solution and cultivated with the first anti-substances, *viz.*, COL5A1, COL1A1, E-cadherin, N-cadherin, snail, GADPH rabbit polyclonal antibody (Proteintech, USA), vimentin, and vinculin mouse polyclonal antibody (Proteintech, USA) at 4°C overnight. Subsequently, the films were washed three times in TBST again, each for 10 min. Goat anti-rabbit IgG H&L (HRP) and goat anti-mouse IgG H&L (HRP) (Abcam, USA) were added then for cultivation. Afterwards, the membranes were cleaned in TBST as per the steps mentioned above to analyze relative protein expression with Image-Pro Plus software 6.0.

### Wound-Healing Assay

When the confluency of cells reached 80%–90%, a 200-μl pipet was employed to scratch along the central axis of the culture dish, which was then washed twice with PBS. Subsequently, the cells were cultivated in an intermediary without serum. Furthermore, wound-healing pictures were harvested under a microscope at 0 and 24 h after the wound occurrence. We measured the migration rate with NIH ImageJ software.

### Transwell Assay

Transwell assay included migration and invasion assays. The migrating cells (4 × 10^5^) were inoculated into a 24-transwell chamber with an 8-µm Matrigel-coated aperture film, and the invading cells (5 × 10^5^) were uniformly inoculated in a 24-well plate containing matrix glue. The upper chamber contained RPMI 1640 intermediary without serum, whereas the lower chamber was filled with RPMI 1640 medium mixed with 10% FBS. Subsequent to the incubation under 37°C for 24 h, the cells on the superficial region of the film were discarded *via* a cotton swab, and those on the lower surface of the membrane were dyed with gentian violet dye after fixation with 75% alcohol. Later, the cells were imaged *via* a microscopic device in order to obtain the images for analysis with NIH ImageJ software.

### CCK-8 Assay

Cells in the log-growth stage (200 µl) were seeded in a 96-well plate at a concentration of 1×10^5^ cells per well and incubated for 24 h. Thereafter, 10 µl of CCK8 reagent was supplemented to all wells, followed by cultivation in a dark incubator for 120 min. Thereafter, the optical density (OD) results of this mixture (cells + reagent) were identified *via* ELISA with a wavelength of 450 nm. The proliferation rate of cells was determined by comparing the OD values of the mixture after inoculation for 6, 24, 48, 72, and 96 h.

### EdU Assay

An EdU assay kit (RiboBio, Guangzhou, China) was adopted for carrying out the EdU assay. Initially, 2 ml of cells were plated into a 96-well dish with a concentration of 4 × 10^4^ cells/well, cultivated with EdU reaction fluid (50 µM) for 2 h, followed by permeabilization with 0.3% TritonX-100 (Jiayan Biotech, Guangzhou, China) for 10 min, and fixation in 4% paraformaldehyde (Leagene, Beijing, China) for 0.5 h. Subsequently, 3% bovine serum albumin (BSA) (Solarbio, Beijing, China) was applied to wash the cells three times, each for 5 min, after which the cells were treated with an Apollo reaction mixture for 0.5 h, before being cleaned three times in 3% BSA, each for 5 min. Subsequently, the nuclei of cells were dyed in DAPI (Solarbio, Beijing, China) for 10 min and examined under the microscope.

### Immunofluorescence

After being cultured, the cells were subjected to fixation in 4% paraformaldehyde (Leagene, China) for 15 min and permeabilization in 0.3% Triton (Jiayan Biotech, China) and finally blocked with 3% BSA (Solarbio, China) for 30 min. Cells were afterwards cultivated with primary antibodies, *viz*., E-cadherin rabbit polyclonal anti-substance 1:100 and vimentin mouse polyclonal antibody 1:200 under 4°C overnight. Subsequent to cleaning, the cells were cultivated with anti-mouse IgG fluorescence-conjugated secondary anti-substance, anti-rabbit IgG fluorescence-conjugated secondary anti-substance (goat anti-mouse IgG H&L, 1:2,000), and goat antirabbit IgG H&L, 1:2,000 (Proteintech, USA) under room temperature for 60 min. Then, the nuclei of cells were stained *via* DAPI (1 µg/ml) (Solarbio, China), which were observed under a fluorescent microscope (DM6 B; Leica Microsystems) at 63× magnification, to analyze the captured images with Fiji software.

### Animal Experiments

All the animal assays were accepted by the GuangXi Medical University Ethical Board on Animal Research. Nude mice with a BAL/c background (6-week-old females with a bodyweight of 16–18 g) from Junke Bioengineering Co., Ltd. (Nanjing, China) were raised and monitored according to requirements for a specific pathogen-free (SPF) status. For the cancer-growth assays, 1 × 10^6^ K1 cells were introduced into BALB/c nude mice *via* injection. The tumorous volume was observed and measured every 3 days throughout the experimental period with Vernier calipers. One week later, tumorous volume was computed *via* the equation: volume = 0.5 × length × width^2^.

Approximately 21 days later, when the cancers reached a mean volume of 100 mm^3^, the animals were stochastically separated into two groups (five mice/group) and were delivered an intratumoral injection containing 20 mg/kg miR-29b-3p agomir or control agomir (Guangzhou, China) every 3 days for 12 days (four injections in total), which was repeated four times. After 33 days, the tumors were collected from the sacrificed mice and weighed; the growth of these xenografts was evaluated after the tumors were removed, weighed, and photographed, with tissues fixed with 4% paraformaldehyde and paraffin-embedded for immunohistochemistry (IHC) analyses.

### Immunohistochemistry

Immunohistochemistry of mouse tumors was performed according to the following standard procedures: tumor tissue embedding, specimen sectioning, dewaxing, rehydration, and antigen recovery. After serum occlusion, sections were incubated with COL1A1 or COL5A1 antibody (1:100, desaturated in PBS with 1% standard goat serum) under 4°C for a single night and afterwards were incubated with second anti-substances under RT for 60 min. The staining procedure was performed following the DAB detection kit protocol with DAB (DAB-0031; Fuzhou Maixin Biotech Company, Fuzhou, China).

### Dual-Luciferase Reporter Assay

In order to examine the straight binding of miR-29b-3p to target genes, COL1A1 and COL5A1, a luciferase reporter assay was completed as previously described. Reporter vectors, namely, Luc-COL1A1 and Luc-COL5A1, or mutation vectors with miR-29b-3p-binding spot mutations, were cotransfected with miR-29b-3p mimetic substances (RiboBio, China) into HEK-293 T cells. Firefly luciferase and Renilla fluorescein enzyme activity were identified following transfection for 48 h with the Dual-Luciferase Reporter Assay System as per the supplier’s specification. Moreover, the miR-29b-3p promotor-reporter construct with wild type was introduced into HEK-293 T cells *via* transfection, and the pTK-Club vector was considered the control, all of which were treated with hypoxic or normoxic conditions for 48 h.

### Quantification and Statistical Analysis

Statistical analyses and the construction of graphs were conducted with statistical packages, namely, GraphPad Prism 5 (USA) and SPSS 21.0 (IBM, USA). The data collected in the experiments are described as x ± SD. A t-test was used for cell testing of intergroup contrast, and Mann–Whitney U-test was utilized for clinical data analysis. A Kruskal–Wallis test was applied when comparing three or more groups. p < 0.05 indicates a remarkable diversity.

## Results

### Downregulation of miR-29b-3p Is Associated With Disease Development in Human PTC

**Figure 1:**
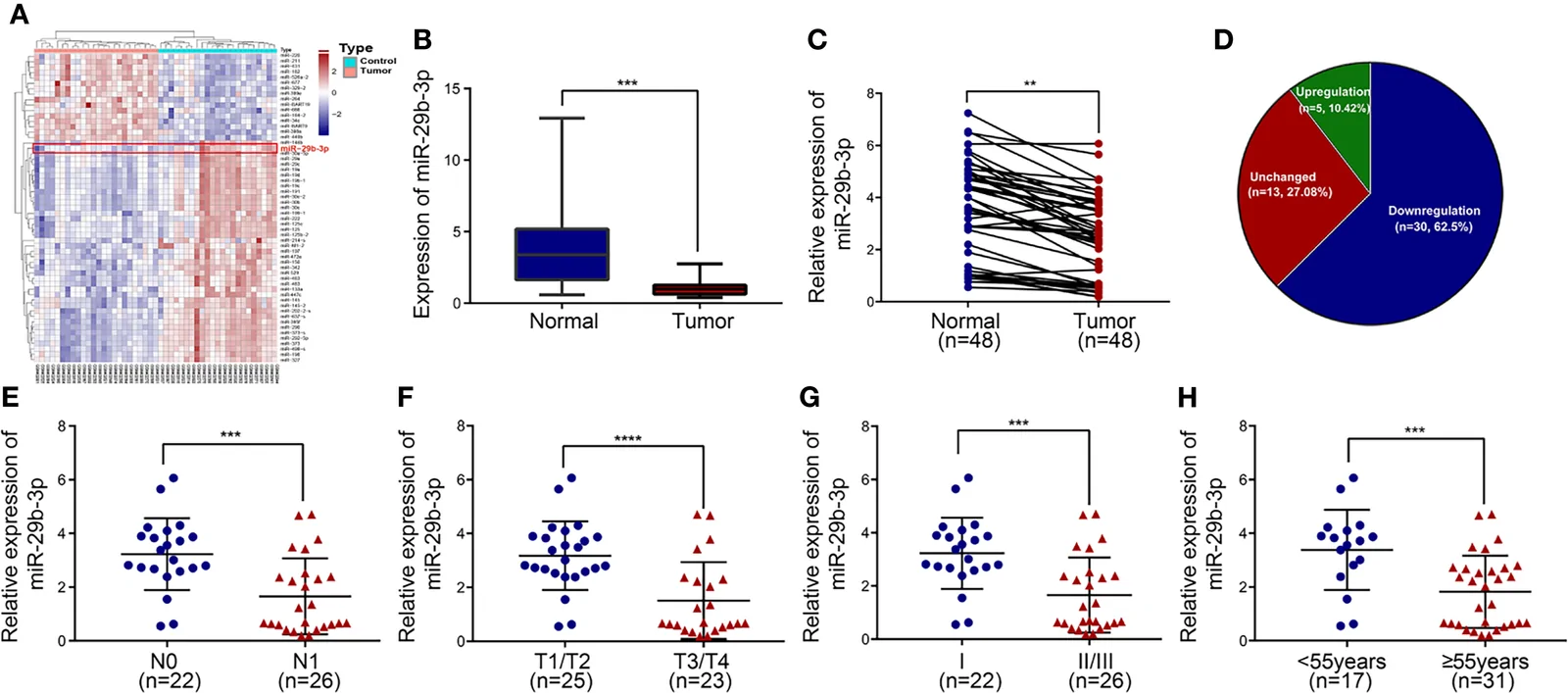
Downregulation of miR-29b-3p is associated with disease progression in PTC. **(A)** Heatmap of differentially expressed genes in GEO dataset coloring the samples groups. **(B)** Expression level of miR-29b-3p in PTC based on data retrieved from the GEO database and the ArrayExpress database. **(C, D)** The expression of miR-29b-3p was evaluated by qRT-PCR in 48 paired human PTC tissues and their matched adjacent non-tumor thyroid tissues. MiR-29b-3p expression was significantly downregulated in PTC tumor tissues compared with the corresponding non-tumor thyroid tissues (U6 was used as an internal control). **(E–H)** Relationships between miR-29b-3p expression and TMN stage of PTC. In the figure, **p < 0.01, ***p < 0.001, ****p < 0.0001.

The expression of miR-29b-3p was much more downregulated in PTC tissues than adjacent normal tissues in GEO databases (GSE15740) (filtered by p < 0.05, **Figures 1A, B**). This result was validated in our 48 pairs of tissue samples by qRT-PCR (**Figure 1C**). Specifically, miR-29b-3p expression was downregulated in 62.5% of samples, while no significant difference was demonstrated in 27.08% of samples and was upregulated in 10.42% of samples (**Figure 1D**). Subsequently, we investigated the association of miR-29b-3p with clinicopathological features, which demonstrated an obvious decrease in miR-29b-3p expression in N1 vs. N0 PTCs, in T3/T4 vs. T1/T2 PTCs, in TNM stage II/III vs. I PTCs, and in age ≥55 years vs. <55 years PTCs (**Figures 1E**–**H**; **Table 2**).

**Table 2:** The relationship between the relative expression of miR-29b-3p and clinicopathological characteristics in thyroid cancer patients.

Clinicopathological	Variable	No	miR-29b-3p	p-Value
Age, years	<55	17	3.383 ± 1.494	0.001*
	≥55	31	1.825 ± 1.348	
Gender	Male	15	1.399 ± 1.632	0.003*
	Female	33	2.821 ± 1.355	
Tumor	T1-T2	25	3.176 ± 1.271	0.000*
	T3-T4	23	1.507 ± 1.427	
Lymph node metastases	N0	22	3.228 ± 1.337	0.000*
	N1	26	1.656 ± 1.413	
TNM tumor stage	I	22	3.228 ± 1.337	0.000*
	II-III	26	1.656 ± 1.413	

*p < 0.05.

### miR-29b-3p Inhibits PTC Cell Proliferation and Metastasis *In Vitro*

**Figure 2:**
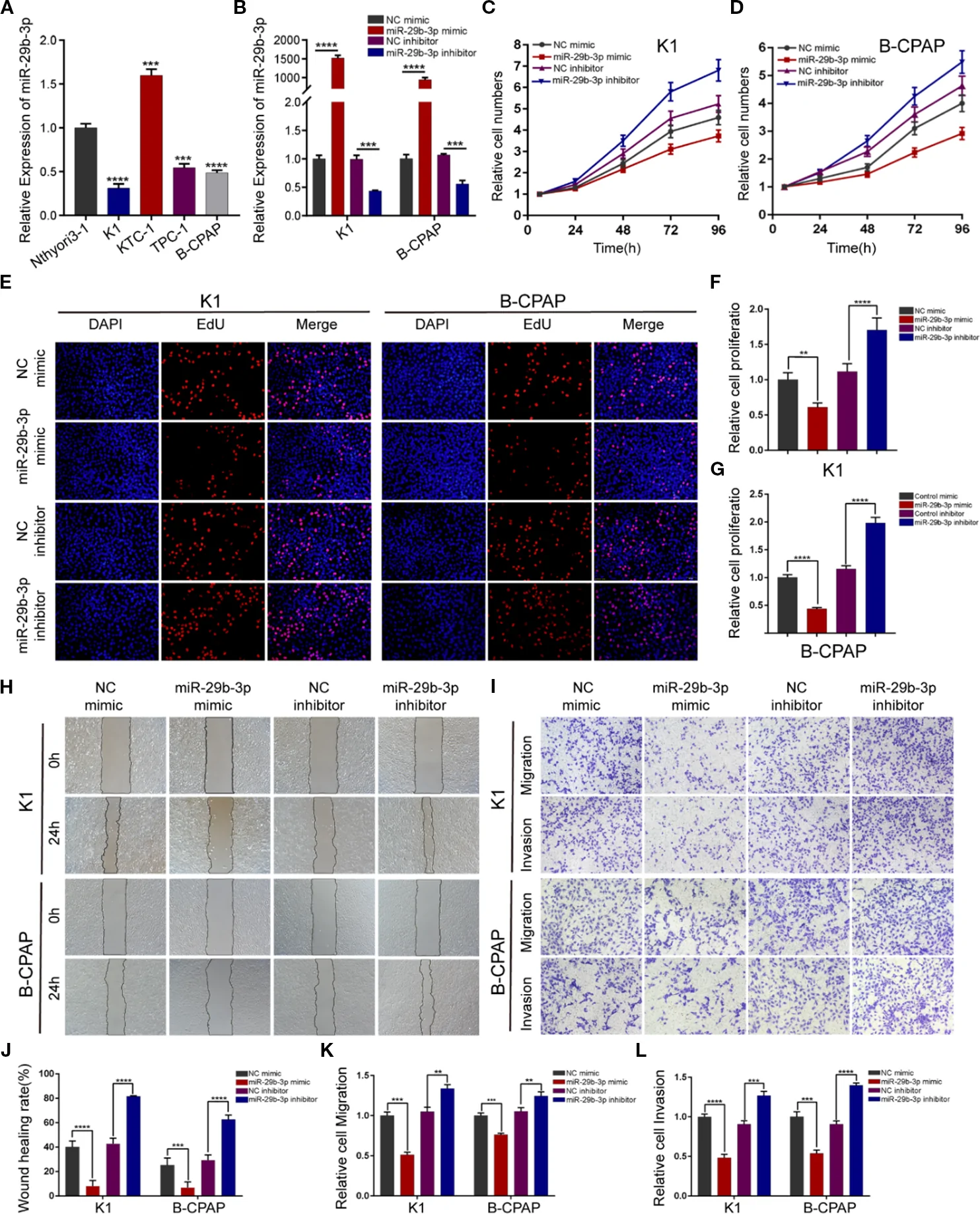
miR-29b-3p inhibits PTC cell migration and invasion *in vitro*. **(A)** Expression of miR-29b-3p was verified by qRT-PCR in PTC cell lines (K1, KTC-1, TPC-1, and B-CPAP) and normal thyroid cell lines (Nthyori3–1); two lowly expressing (K1 and B-CPAP) cell lines were selected for further study. **(B)** The knockdown and overexpression efficiencies of miR-29b-3p was verified by qRT-PCR after transfection with miR-29b-3p mimic/inhibitor or NC mimic/inhibitor. Cell proliferation ability of PTC cells was determined by CCK-8 assays **(C, D)** and EdU assays **(E–G)** after miR-29b-3p overexpression or knockdown. The effects of miR-29b-3p overexpression and knockdown in PTC cells on cell migration and invasion were analyzed by wound healing assays **(H)** and Transwell assays **(I)**. **(J, L)** Results of the Transwell assays and wound healing assays. In the figure,**p < 0.01, ***p < 0.001,****p < 0.0001.

To understand the mechanism involved in the downregulation of miR-29b-3p in PTC, its expression levels of miR-29b-3p were examined, employing qRT-PCR in the normal thyroid cell line (Nthyori3-1) and 4 PTC cell lines (TPC-1, KTC-1, K1, and B-CPAP). Among these PTC cell lines, the K1 and the B-CPAP cells showed the lowest expression of miR-29b-3p. Thus, these two cell lines were therefore selected for the follow-up experiments (**Figure 2A**).

To explore the function of miR-29b-3p in PTC cells, K1 and B-CPAP cells were transfected with miR-29b-3p mimic or miR-29b-3p inhibitor, NC mimic and NC inhibitor were transfected as respective controls; and transfection efficiency was evaluated by qRT-PCR. It was observed that the expression level of miR-29b-3p significantly increased in PTC cells after transfection with miR-29b-3p mimic as compared to that in cells transfected with NC mimic. In addition, the expression of miR-29b-3p significantly decreased after transfection of the cells with miR-29b-3p inhibitor (**Figure 2B**).

CCK-8 and EdU assays revealed that, when transfected with miR-29b-3p mimic, the proliferation ability of PTC cells was reduced significantly in comparison to those transfected with NC mimic. In turn, the downregulated expression of miR-29b-3p contributed to the proliferation of PTC cells (**Figures 2C**–**E**; **Supplementary Table S2**). Accordingly, the results were quantified and are presented in **Figures 2F**–**G** (all p-values <0.01).

Furthermore, the PTC cells’ migration and invasive ability were assessed using Transwell and wound-healing assays (48 h after transfection for K1 and the B-CPAP cells). The results suggested that overexpression of miR-29b-3p inhibited proliferation and migration abilities, while an opposite effect occurred in the miR-29b-3p-knockdown cells; the results are presented in **Figures 2H**–**I**. The quantification of these results is shown in **Figures 2J**–**L** and **Supplementary Tables S3** and **S4** (**p < 0.01 and *** p < 0.001; **** p < 0.0001).

### miR-29b-3p Directly Targets COL1A1 and COL5A1

**Figure 3:**
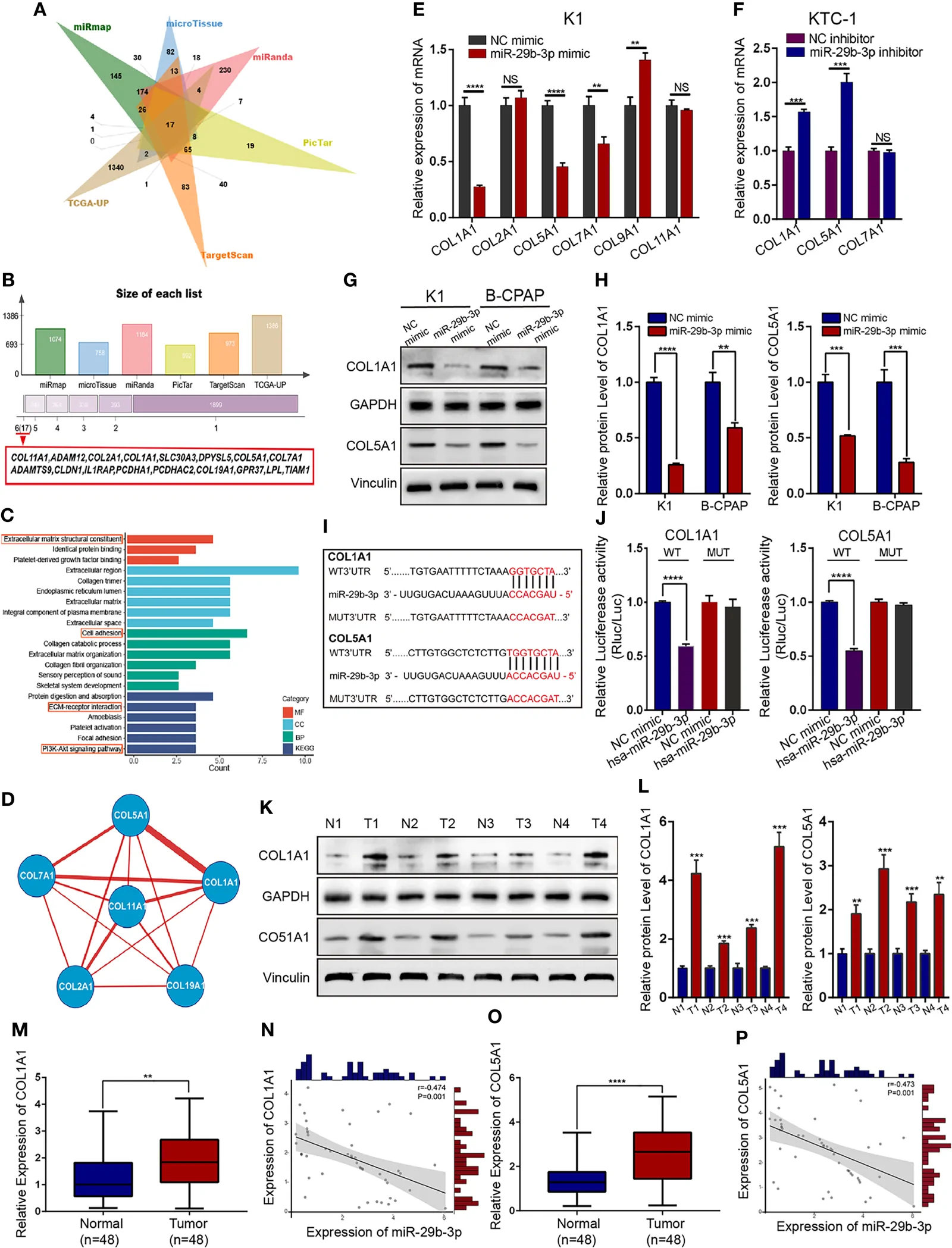
COL1A1 and COL5A1 as direct targets of miR-29b-3p in PTC tissues. **(A, B)** The bioinformatic predictions of miR-29b-3p candidate target genes and Venn diagram depicting the overlap of target genes predicted by five miRNA databases (miRmap, microTissue, miRanda, PicTar, and TargetScan) and TCGA datasets. **(C)** GO and KEGG enrichment analysis of candidate miR-29b-3p target genes. **(D)** The miR-target gene regulatory network constructed using Cytoscape. **(E, F)** Fold change in mRNA expression of six candidate target genes upon transfection of miR-29b-3p mimic/inhibitor and validation of COL1A1 and COL5A1 as the direct target genes of miR-29b-3p by qRT-PCR. **(G, H)** Western blot analysis of COL1A1 and COL5A1 expression after transfection with miR-29b-3p mimics. **(I, J)** The 3-UTRs of COL1A1 and COL5A1 as potential binding sites for miR-29b-3p, determined by the luciferase assays. (**K, L**) Western blots showing the relative protein expression levels of COL1A1 and COL5A1 in PTC tissues and adjacent non-cancerous normal tissues from four patients. **(M–P)** Relative expression levels of COL1A1 and COL5A1 in 48 paired human PTC tissues and adjacent non-tumor thyroid tissues, as evaluated by qRT-PCR. Both COL1A1 and COL5A1 mRNA highly expressed in PTC tissues and negatively correlated with miR-29b-3p expression. In the figure, **p < 0.01, ***p < 0.001, ****p < 0.0001; NS, not significant.

Using online software, we predicted potential targets of miR-29b-3p, and 17 genes were found to be potential targets (**Figures 3A, B**). GO function annotations and KEGG pathway analysis were carried out for the target genes. The results revealed that the potential target genes are involved in the regulation of ECM adhesion dynamics (**Figure 3C**). The PPI network data, with the confidence score set at 0.4, revealed that the hub genes bearing higher degrees comprised COL1A1, COL2A1, COL5A1, COL7A1, COL11A, and COL9A1 (**Figure 3D**). Based on the above results, we further validated the relationship between hub genes and miR-29b-3p. Specifically, we performed qRT-PCR to detect the expression of hub genes at mRNA levels in PTC cells transfected with miR-29b-3p mimic or inhibitor. Both COL1A1 and COL5A1 showed significant inverse correlations with miR-29b-3p expression in PTC cells (**Figures 3E, F**). Western blot analysis results showed that miR-29b-3p overexpression obviously suppressed the protein expression of COL1A1 and COL5A1 in PTC cells (**Figures 3G, H**; **Supplementary Table S5**).

To test whether COL1A1 and COL5A1 have a transcriptional regulatory relationship, we separately overexpressed COL1A1 and COL5A1 in order to determine whether the overexpression of COL1A1 and COL5A1 would bear an influence on COL5A1 and COL1A1 expression in PTC cells, and accordingly, qRT-PCR analysis results demonstrated no transcriptional regulatory relationship between COL1A1 and COL5A1.

Hence, a dual‐luciferase reporter assay was employed for further verification of the targeted relationship between miR-29b-3p and COL1A1/COL5A1. For this investigation, 3′-UTRs of these two genes were initially cloned downstream of firefly luciferase. Thereafter, the luciferase reporters were co-transfected into HEK-293 T cells with miR-29B-3p mimics. We observed that overexpression of miR-29b-3p significantly reduced luciferase activity in vectors containing COL1A1 and COL5A1 3′-UTR. Moreover, a mutation in the seed regions within 3′-UTRs of either COL1A1 or COL5A1 blocked the inhibition of these genes by miR-29b-3p (**Figures 3I, J**; **Supplementary Table S6**).

The mRNA and protein expression levels of COL1A1 and COL5A1 in PTC tissues and adjacent normal tissues were determined by qRT-PCR and WB analysis. We determined that COL1A1 and COL5A1 were upregulated in PTC tissues and inversely correlated with miR-29b-3p expression (**Figures 3K**–**P**; **Supplementary Table S1**). From these results, it could be inferred that miR-29b-3p downregulated COL1A1 and COL5A1 by means of directly targeting their 3′-UTRs.

### Overexpression of COL1A1 or COL5A1 Partially Blocked the Inhibitive Effects of miR-29b-3p on Cell Invasion

**Figure 4:**
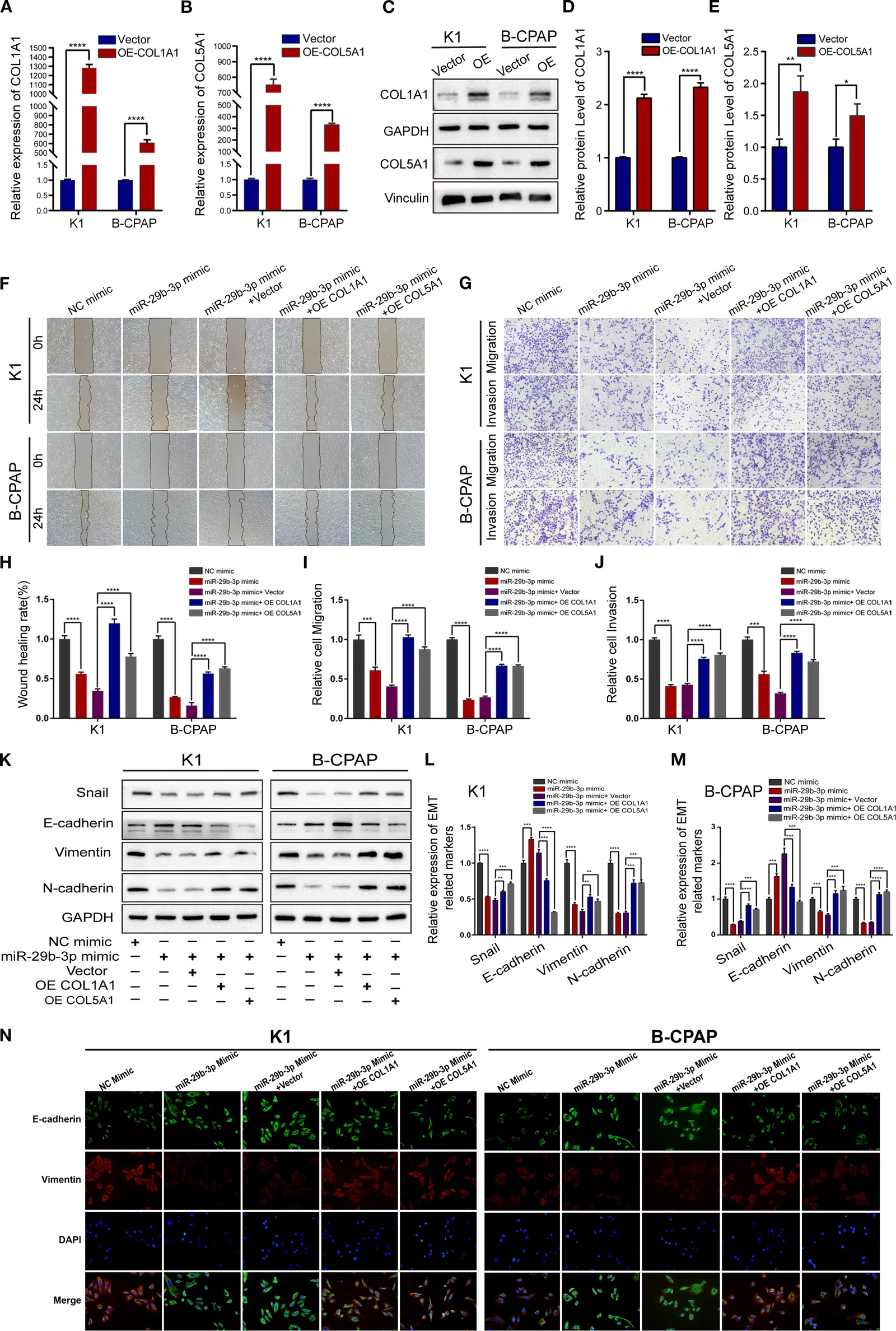
Overexpression of COL1A1 or COL5A1 partially blocks the suppressive effects of miR-29b-3p on invasion in PTC cells. **(A, B)** qRT-PCR and **(C)** Western blot were performed to assess the transfection efficiency of COL1A1 and COL5A1 overexpression plasmids. **(D, E)** Quantification of COL1A1 and COL5A1 levels in Western blot normalized over GAPDH and Vinculin. The wound healing assays **(F)** and Transwell assays **(G)** demonstrated that the overexpression of COL1A1 and COL5A1 could efficiently alleviate the miR-29b-3p mimic-induced inhibition of the migration and invasion activities of K1 and B-CPAP cells. **(H–J)** Quantified results of the wound-healing assays and Transwell assays. Inhibition of miR-29b-3p mimic-induced EMT by overexpressed COL1A1 or COL5A1; the EMT-related proteins were investigated by **(K)** Western blot and **(N)** immunofluorescence. **(L, M)** The EMT-related proteins expression for each group was analyzed by Image J software. In the figure, *p < 0.05, **p < 0.01, ***p < 0.001, ****p < 0.0001.

To investigate whether the proliferation and migration suppressive effect of miR-29b-3p on PTC cells was mediated by repression of COL1A1 or COL5A1, K1 and B-CPAP cells were transfected with COL1A1 or COL5A1 overexpression plasmid, while the control group was transfected with empty vector plasmids. The overexpression efficiency of constructed plasmids was validated by qRT-PCR (**Figures 4A, B**) and Western blot (**Figures 4C**–**E**; **Supplementary Table S7**). Afterwards, PTC cells were transfected with NC mimic, miR-29b-3p mimic, miR-29b-3p mimic + Vector, miR-29b-3p mimic + OE- COL1A1, or miR-29b-3p mimic + OE- COL5A1, respectively. Furthermore, the invasion assay indicated that overexpression of miR-29b-3p inhibits invasion and migration of PTC cells. However, the co-transfection of miR-29b-3p mimic and OE-COL1A1 or OE-COL5A1 partially restored the ability of cells to invade (**Figures 4F, G**; **Supplementary Tables S8, S9**). Experiments were repeated independently, and the results were quantified (**Figures 4H**–**J**). Nevertheless, this result was not confirmed in cell proliferation experiments. Based on the above analysis, we hypothesized that COL1A1 and COL5A1 might be mainly involved in the proliferation and invasion of PTC cells.

The tumor invasion and metastatic potential were enhanced by the epithelial–mesenchymal transition (EMT). We examined whether miR-29b-3p, COL1A1, and COL5A1 promote EMT; in indicated cells after the same transfection, Western blot detected EMT‐related proteins including E-cadherin, Snail, Vimentin, and N-cadherin. After treatment with the miR-29b-3p mimic, the results indicated that the expression of E-cadherin was upregulated in the K1 and B-CPAP cells. In contrast, the expressions of Snail, Vimentin, and N-cadherin were downregulated. These results established that miR-29b-3p inhibited the EMT process. However, the results were reversed after transfection of the K1 and B-CPAP cells with a plasmid containing either of the genes-COL1A1 or COL5A1 (**Figures 4K**–**M**; **Supplementary Table S10**). Additionally, the immunofluorescence method was used to obtain the same results as WB (**Figure 4N**). Collectively, these results demonstrated that miR-29b-3p regulated cell migration, invasion, and EMT in PTC cells by targeting COL1A1 and COL5A1.

### Downregulating COL1A1 or COL5A1 Expression Can Inhibit Migration, Invasion, and EMT in PTC Cells

**Figure 5:**
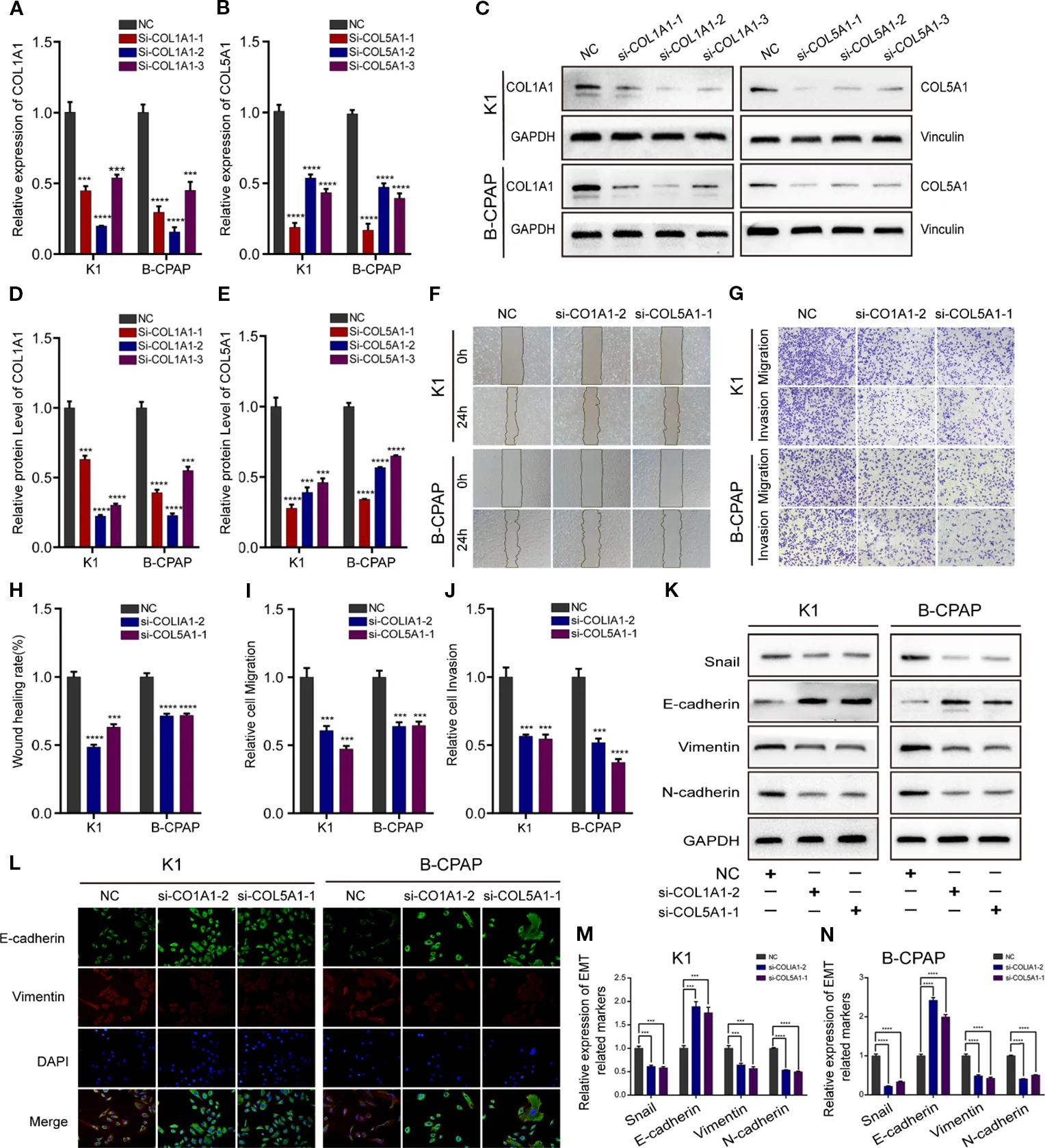
Downregulating COL1A1 or COL5A1 expression can inhibit migration, invasion, and EMT in PTC cells. The knockdown efficiency of COL1A1 and COL5A1 were analyzed by **(A, B)** qRT-PCR and **(C–E)** and Western blotting. Additionally, **(F)** wound healing and **(G)** Transwell assays were conducted to evaluate PTC cells migration and invasion, which proved that both si-COL1A1–2 and si-COL5A1–1 significantly impaired migration and invasion ability in K1 and B-CPAP cells. **(H–J)** The statistical results of wound healing assays and Transwell assays. **(K)** Western blot and **(L)** immunofluorescence analyses were performed to determine the expression of EMT-related proteins detection in PTC cells transfected with si-COL1A1–2 and si-COL5A1–1. **(M, N)** Western blotting assay showing the protein expression levels of EMT-related in PTC cells. In the figure, ***p < 0.001, ****p < 0.0001.

To further verify the involvement of COL1A1 and COL5A1 in migration and invasion of PTC cells, siRNA knockdown was performed. The efficiency of siRNA knockdown was verified by qRT-PCR and Western blot. According to our results, si-COL1A1–2 and si-COL5A1–1 demonstrated the most significant knockdown efficiency, which in turn showed a capacity to reduce the expression of COL1A1 and COL5A1 mRNA and protein by 60%–80% (**Figures 5A**–**C**; **Supplementary Table S7**) and so was used in the subsequent experiments. Western blot quantification are shown in **Figures 5D, E**. Wound healing and Transwell assays confirmed that silencing of COL1A1 or COL5A1 markedly inhibited the invasive capacity of K1 and B-CPAP (**Figures 5F, G**). The statistical analyses of the results mentioned above are represented in **Figures 5H**–**J** and **Supplementary Tables S11** and **S12**. In addition, the expression of EMT-related proteins was detected by Western blot. After the analysis, it was observed that after transfection with si-COL1A1–2 or si-COL5A1–1, the protein expression of E-cadherin was upregulated, whereas the protein expressions of Snail, Vimentin, and N-cadherin were significantly downregulated (**Figure 5K**). These data were also confirmed through immunofluorescence analysis (**Figure 5L**). Quantification of WB analysis is shown in **Figures 5M, N** and **Supplementary Table S13**.

### miR-29b-3p Inhibits PTC Cell Growth and Decreases the Expression of COL1A1 and COL5A1 *In Vivo*

**Figure 6:**
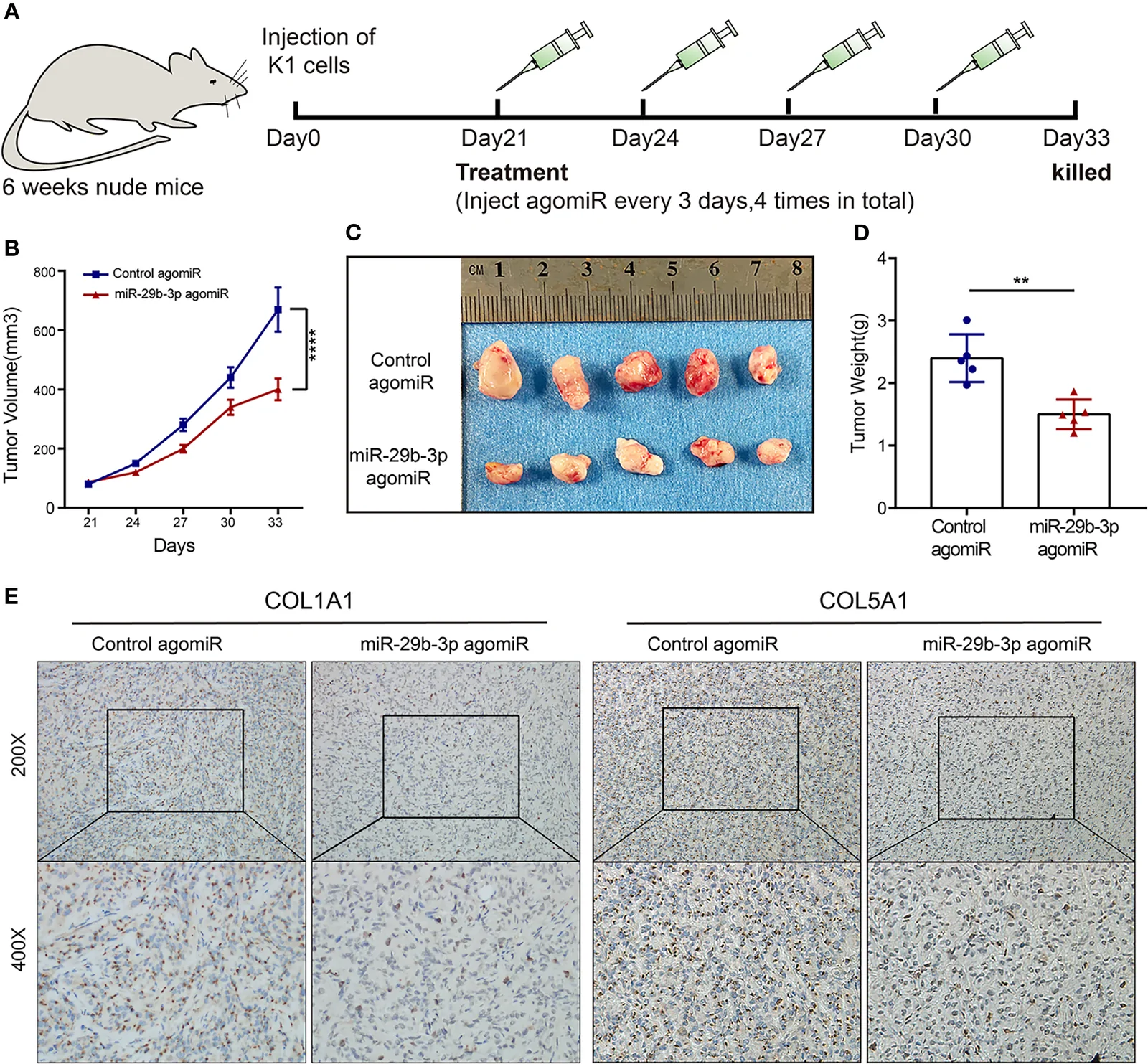
miR-29b-3p inhibits PTC cell growth and decreased COL1A1 and COL5A1 expression *in vivo*. **(A)** Schematic diagram of the *in vivo* experimental process. **(B)** Tumor growth curves of control agomiR (n = 5) and miR-29b-3p agomiR (n = 5) in nude mice. **(C)** Gross pathology of tumors at post-transplanted day 33. **(D)** Statistical analysis of the mean tumor weight of tumor xenografts from nude mice after 33 days of treatment. **(E)** Immunohistochemistry analysis of protein expression levels of COL1A1 and COL5A1 in tumor xenografts treated with miR-29b-3p agomiR and control agomiR. In the figure, **p < 0.01, ****p < 0.0001.

A tumor formation assay was performed in nude mice to confirm the effects of miR-29b-3p on the tumorigenesis of PTC. We successfully established a subcutaneously implanted tumor model of PTC in nude mice by subcutaneous injection of K1 cells. After 3 weeks’ growth, miR-29b-3p agomir or control agomir were intratumorally injected every 3 days for 12 days, and tumor growth was monitored (**Figure 6A**). According to the curves of tumor growth, an injection of the miR-29b-3p agomir significantly inhibited tumor growth compared to the control group (**Figure 6B**; **Supplementary Table S14**). As shown in **Figures 6C, D**, 33 days after tumor xenografts, tumors were excised from the nude mice and weighed, and tumors in the miR-29b-3p agomir group were lighter than the control agomir group. Furthermore, immunohistochemical analysis of COL1A1 and COL5A1 expression was performed using the xenograft tumors from miR-29b-3p agomir or control agomir-treated mice. It was revealed that the protein expression levels of COL1A1 and COL5A1 were significantly decreased in the miR-29b-3p agomir group as compared with that of the control agomir group (**Figure 6E**). The results of our *in vivo* experiments established a negative correlation between miR-29b-3p and COL1A1 and between miR-29b-3p and COL5A1.

## Discussion

It is critical to explore molecular biomarkers of invasion and proliferation for prognosis and for carrying out personalized treatment regimens of tumor (23–27). MiRNA is a component of the gene regulation network, which regulates 30% human genes at the post-transcription level and participates in the occurrence of a range of diseases (28). MiRNA dysregulation has been identified as a hallmark of human malignancies (10, 29) and may be correlated with clinicopathological characteristics and disease activity of cancer (30–32). Several studies have demonstrated that miRNAs play an important role in the metastasis and progression of thyroid cancer (33–39). In the present study, we showed for the first time that miR-29b-3p expression was downregulated in PTC tissues as compared to normal tissues and was significantly negatively correlated in terms of tumor size, local lymph node metastasis, and TNM stage in patients with PTC.

To further explore the biological function of miR-29b-3p in PTC, we conducted experiments *in vivo* and *in vitro*. Further functional analysis revealed that the overexpression of miR-29b-3p could prominently constrain cellular proliferative, migratory, and invasive abilities of PTC cells. Furthermore, *in vivo* experiments showed that miR-29b-3p could inhibit *in vivo* growth of PTC cells in nude mice. This result is also found in other tumors in the study. For instance, miR-29b-3p functioned as an anti-cancer agent in glioblastoma tumors (40). Zhao et al. suggested that miR-29b-3p might inhibit pancreatic ductal adenocarcinoma cell migration, invasion, and endothelial dysfunction tube formation through inhibiting the expression of VGEFA, a well-known pro-cancerous gene (41). Subsequent studies supported that miR-29b-3p could suppress the growth of multiple myeloma and ovarian cancer cells (42, 43) along with the migratory and invasive abilities of liver carcinoma and esophageal cancer cells (44, 45) and the progression of bladder cancer by inhibiting the proliferative, migratory, and invasive abilities of oncocytes (46). These discoveries indicate the common essential function of miR-29b-3p in tumor metastasis and proliferation. The anticancer properties of miR-29b-3p have attracted growing interest, which has prompted us to further explore the downstream target of miR-29b-3p.

MiRNAs regulate gene expression by combining with the mRNA 3′-UTR, leading to target mRNA translation inhibition or cleavage. To explore the biological functions of miR-29b-3p, we found, by online bioinformatic tools, that six collagen genes were the potential target genes of miR-29b-3p. Collagens are extracellular macromolecules that are major structural proteins of the ECM. ECM is a major component of the cell microenvironment and provides support for surrounding cells (47). It plays an essential role in regulating cell proliferation, migration, morphology, function, and development (48). ECM remodeling is a critical biological process for the adhesion, motility, and migration of tumor cells (49, 50). Studies have suggested that ECM participates in tumorigenesis through cellular migration, growth, and EMT (51, 52). It is noteworthy that collagen-containing fibers can promote the migratory ability of oncocytes, and their proliferation and migration can be fostered by increasing the collagen density (53). Therefore, increasing evidence confirms that collagen is an essential regulatory protein that controls tumor infiltration, angiogenesis, invasion, and migration (54–57). Moreover, collagens have also been demonstrated to be involved in the initiation and progression of thyroid cancer (58–60).

In our study, the data from qRT-PCR, WB, and dual luciferase reporter assay solidly confirmed that miR-29b-3p directly targeted COL1A1 and COL5A1 3′‐UTR and thus caused degradation of type I and V collagens, which could explain the downregulation of miR-29b-3p in clinical tumor tissues. Our findings showed that COL1A1 and COL5A1 are highly expressed at both mRNA and protein levels in PTC tissues. Type I collagen is an important constituent of ECM and is the most abundant member of the collagen family. It has also been confirmed that COL1A1 can hasten the malignant phenotype and progression of various malignancies, such as gastric cancer, prostate carcinoma, mammary carcinoma, pancreatic ductal carcinoma, liver carcinoma, and lung carcinoma (61–67). A substantial number of studies have verified that COL5A1 is an essential factor for the metastasis of cells in gastric cancer (68), which may also be a new prognostic factor with respect to lung adenocarcinoma, breast cancer, tongue squamous cell carcinoma, and other tumors (69–71). High expression of COL5A1 can also accelerate the growth and progression of renal cell carcinoma (72). The functional analysis unveiled that both COL1A1 and COL5A1 facilitated the migratory and invasive abilities of PTC cells, and their overexpression could reverse the inhibitory roles of miR-29b-3p in the malignant behavior of PTC cells. We have further demonstrated that COL1A1 and COL5A1 trigger the EMT process and therefore participate in the maintenance of the aggressive phenotype and migratory phenotype of PTC cells. Nevertheless, in contrast to the controls, the overexpression of COL1A1 or COL5A1 did not markedly change the proliferative ability of PTC cells. Our study is the first to prove that both COL1A1 and COL5A1 enhance the invasion, migration, and EMT of PTC cells. Nevertheless, more evidence is essential to explain the pathways of these target genes participating in the migratory and invasive activities of PTC cells.

The above indicates that miR-29b-3p participates in the development of PTC by the remodeling of the ECM, which provides a new theoretical basis for future studies to PTC tumor microenvironment and its progression. However, several deficiencies exist in this study, as we observed a consistent relationship between the COL1A1 and COL5A1, while no regulatory relationship between them at the transcriptional level was shown. These results reflected that COL1A1 and COL5A1 were mainly regulated at the protein–protein level, not at the transcriptional level. However, due to the limitations in experimental conditions, the relationship of protein–protein interaction could not be investigated in this study. In addition, the ideal tumor metastatic models of PTC were not successfully established through injecting K1or B-CPAP cells into the abdomen or tail vein in this study.

In conclusion, we identified that miR-29b-3p is significantly downregulated in PTC, being correlated with cell proliferation, migration, and invasion. The mechanistic investigation revealed that miR-29b-3p directly targeted COL1A1 and COL5A1, thereby suppressing ECM remodeling, which is crucial for tumor invasion and metastasis. This study also generates new ideas for risk assessment and miRNA replacement therapy in PTC.

## Data Availability Statement

The datasets presented in this study can be found in online repositories. The names of the repository/repositories and accession number(s) can be found in the article/**Supplementary Material**.

## Ethics Statement

The studies involving human participants were reviewed and approved by the Bioethics Committee of First Affiliated Hospital of Guangxi Medical University [No. 2015(KY-E-018)]. The patients/participants provided their written informed consent to participate in this study. The animal study was reviewed and approved by the Animal Ethics Committee of Guangxi Medical University (No. 202010015).

## Author Contributions

CW, YW, and JC contributed to the study conception and design. Material preparation, data collection, and analysis were performed by WH, ZY, SZ, JW, ZF, KZ, and SL. The first draft of the manuscript was written by CW. JC contributed to checking of this manuscript. All authors contributed to the article and approved the submitted version.

## Funding

This work was supported by the National Natural Science Foundation of China (grant no. 82060430), the Guangxi Science and Technology Project (grant no. AD19245196), the Guangxi Key Research and Development Project (grant no. AB18126058), and the Guangxi Scientific Research and Technology Development Project (grant no. 1598011-4).

## Conflict of Interest

The authors declare that the research was conducted in the absence of any commercial or financial relationships that could be construed as a potential conflict of interest.

## Publisher’s Note

All claims expressed in this article are solely those of the authors and do not necessarily represent those of their affiliated organizations, or those of the publisher, the editors and the reviewers. Any product that may be evaluated in this article, or claim that may be made by its manufacturer, is not guaranteed or endorsed by the publisher.

## References

[1] Siegel R Miller K Fuchs H Jemal A. Cancer Statistics, 2021. CA: Cancer J Clin (2021) 71:7–33. doi: 10.3322/caac.21654, PMID: 33433946

[2] Mao Y Xing M. Recent Incidences and Differential Trends of Thyroid Cancer in the USA. Endocr Relat Cancer (2016) 23:313–22. doi: 10.1530/erc-15-0445, PMID: 26917552 PMC4891202

[3] LiVolsi V. Papillary Thyroid Carcinoma: An Update. Mod Pathol (2011) 24:S1–9. doi: 10.1038/modpathol.2010.129, PMID: 21455196

[4] Na K Choi H. Immune Landscape of Papillary Thyroid Cancer and Immunotherapeutic Implications. Endocr Relat Cancer (2018) 25:523–31. doi: 10.1530/erc-17-0532, PMID: 29507047

[5] Hartl D Travagli J. The Updated American Thyroid Association Guidelines for Management of Thyroid Nodules and Differentiated Thyroid Cancer: A Surgical Perspective. Thyroid (2009) 19:1149–51. doi: 10.1089/thy.2009.1600, PMID: 19888857

[6] Roman B Brito J Saucke M Lohia S Jensen C Zaborek N. National Survey of Endocrinol Ogists and Surgeons Regarding Active Surveillance for Low-Risk Papillary Thyroid Cancer. Endocr Pract (2021) 27:1–7. doi: 10.1016/j.eprac.2020.11.003, PMID: 33471727 PMC8185804

[7] Lee S Roh J Gong G Cho K Choi S Nam S. Risk Factors for Recurrence After Treatment of N1b Papillary Thyroid Carcinoma. Ann Surg (2019) 269:966–71. doi: 10.1097/sla.0000000000002710, PMID: 29462007

[8] Haugen B Alexander E Bible K Doherty G Mandel S Nikiforov Y. 2015 American Thyroid Association Management Guidelines for Adult Patients With Thyroid Nodules and Differentiated Thyroid Cancer: The American Thyroid Association Guidelines Task Force on Thyroid Nodules and Differentiated Thyroid Cancer. Thyroid (2016) 26:1–133. doi: 10.1089/thy.2015.0020, PMID: 26462967 PMC4739132

[9] Chen K Rajewsky N. The Evolution of Gene Regulation by Transcription Factors and microRNAs. Nat Rev Genet (2007) 8:93–103. doi: 10.1038/nrg1990, PMID: 17230196

[10] Iorio M Croce C. Causes and Consequences of microRNA Dysregulation. Cancer J (Sudbury Mass) (2012) 18:215–22. doi: 10.1097/PPO.0b013e318250c001, PMID: 22647357 PMC3528102

[11] Rupaimoole R Slack F. MicroRNA Therapeutics: Towards a New Era for the Management of Cancer and Other Diseases. Nat Rev Drug Discov (2017) 16:203–22. doi: 10.1038/nrd.2016.246, PMID: 28209991

[12] Kasinski A Slack F. Epigenetics and Genetics. MicroRNAs En Route to the Clinic: Progress in Validating and Targeting microRNAs for Cancer Therapy. Nat Rev Cancer (2011) 11:849–64. doi: 10.1038/nrc3166, PMID: 22113163 PMC4314215

[13] Hao C Xu C Zhao X Luo J Wang G Zhao L. Up-Regulation of VANGL1 by IGF2BPs and miR-29b-3p Attenuates the Detrimental Effect of Irradiation on Lung Adenocarcinoma. J Exp Clin Cancer Res CR (2020) 39:256. doi: 10.1186/s13046-020-01772-y, PMID: 33228740 PMC7687693

[14] Worst T Previti C Nitschke K Diessl N Gross J Hoffmann L. miR-10a-5p and miR-29b-3p as Extracellular Vesicle-Associated Prostate Cancer Detection Markers. Cancers (2019) 12:43. doi: 10.3390/cancers12010043, PMID: 31877768 PMC7017198

[15] Hozaka Y Seki N Tanaka T Asai S Moriya S Idichi T. miR-29-3pmolecular Pathogenesis and Regulation of the -Family: Involvement of and in Intra-Hepatic Cholangiocarcinoma. Cancers (2021) 13:2804. doi: 10.3390/cancers13112804, PMID: 34199886 PMC8200054

[16] Sun Y Wang P Yang W Shan Y Zhang Q Wu H. The Role of lncRNA MSC-AS1/miR-29b-3p Axis-Mediated CDK14 Modulation in Pancreatic Cancer Proliferation and Gemcitabine-Induced Apoptosis. Cancer Biol Ther (2019) 20:729–39. doi: 10.1080/15384047.2018.1529121, PMID: 30915884 PMC6605982

[17] Lv M Zhong Z Huang M Tian Q Jiang R Chen J. lncRNA H19 Regulates Epithelial-Mesenchymal Transition and Metastasis of Bladder Cancer by miR-29b-3p as Competing Endogenous RNA. Biochim Biophys Acta Mol Cell Res (2017) 1864:1887–99. doi: 10.1016/j.bbamcr.2017.08.001, PMID: 28779971

[18] Karamanos N Piperigkou Z Passi A Götte M Rousselle P Vlodavsky I. Extracellular Matrix-Based Cancer Targeting. Trends Mol Med (2021) 10:1000–13. doi: 10.1016/j.molmed.2021.07.009, PMID: 34389240

[19] Arseni L Lombardi A Orioli D. From Structure to Phenotype: Impact of Collagen Alterations on Human Health. Int J Mol Sci (2018) 19:1407. doi: 10.3390/ijms19051407, PMID: 29738498 PMC5983607

[20] Liang JN Zou X Fang XH Xu JD Xiao Z Zhu JN. The Smad3-miR-29b/miR-29c axis mediates the protective effect of macrophage migration inhibitory factor against cardiac fibrosis. Biochim Biophys Acta Mol Basis Dis (2019) 1865:2441–2450. doi: 10.1016/j.bbadis.2019.06.004, PMID: 31175931

[21] Zhao B Song X Guan H. CircACAP2 Promotes Breast Cancer Proliferation and Metastasis by Targeting miR-29a/B-3p-COL5A1 Axis. Life Sci (2020) 244:117179. doi: 10.1016/j.lfs.2019.117179, PMID: 31863774

[22] Cao W Feng Y. LncRNA XIST Promotes Extracellular Matrix Synthesis, Proliferation and Migration by Targeting miR-29b-3p/COL1A1 in Human Skin Fibroblasts After Thermal Injury. Biol Res (2019) 52:52. doi: 10.1186/s40659-019-0260-5, PMID: 31540582 PMC6754631

[23] Marcu L. Imaging Biomarkers of Tumour Proliferation and Invasion for Personalised Lung Cancer Therapy. J Pers Med (2020) 10:222. doi: 10.3390/jpm10040222, PMID: 33198090 PMC7711676

[24] Jiang C Li X Zhao H Liu H. Long Non-Coding RNAs: Potential New Biomarkers for Predicting Tumor Invasion and Metastasis. Mol Cancer (2016) 15:62. doi: 10.1186/s12943-016-0545-z, PMID: 27686732 PMC5043609

[25] Awasthee N Rai V Chava S Nallasamy P Kunnumakkara A Bishayee A. Targeting Iκappab Kinases for Cancer Therapy. Semin Cancer Biol (2019) 56:12–24. doi: 10.1016/j.semcancer.2018.02.007, PMID: 29486318 PMC6108957

[26] Sharma G Okada Y Von Hoff D Goel A. Non-Coding RNA Biomarkers in Pancreatic Ductal Adenocarcinoma. Semin Cancer Biol (2020) 75:153–68. doi: 10.1016/j.semcancer.2020.10.001, PMID: 33049362 PMC8035348

[27] Yu L Xu J Liu J Zhang H Sun C Wang Q. The Novel Chromatin Architectural Regulator SND1 Promotes Glioma Proliferation and Invasion and Predicts the Prognosis of Patients. Neuro-Oncology (2019) 21:742–54. doi: 10.1093/neuonc/noz038, PMID: 30753603 PMC6556862

[28] Tonevitsky A Maltseva D Abbasi A Samatov T Sakharov D Shkurnikov M. Dynamically Regulated miRNA-mRNA Networks Revealed by Exercise. BMC Physiol (2013) 13:9. doi: 10.1186/1472-6793-13-9, PMID: 24219008 PMC3681679

[29] Croce C. Causes and Consequences of microRNA Dysregulation in Cancer. Nat Rev Genet (2009) 10:704–14. doi: 10.1038/nrg2634, PMID: 19763153 PMC3467096

[30] Cui X Liu Y Sun W Ding J Bo X Wang H. Comprehensive Analysis of miRNA-Gene Regulatory Network With Clinical Significance in Human Cancers. Sci China Life Sci (2020) 63:1201–12. doi: 10.1007/s11427-019-9667-0, PMID: 32170623

[31] Fortis S Vaxevanis C Mahaira L Sofopoulos M Sotiriadou N Dinou A. Serum miRNA-Based Distinct Clusters Define Three Groups of Breast Cancer Patients With Different Clinicopathological and Immune Characteristics. Cancer Immunol Immunother CII (2019) 68:57–70. doi: 10.1007/s00262-018-2252-7, PMID: 30276443 PMC11028120

[32] Chen F Li X Fu D Huang J Yang S. Clinical Potential of miRNA-221 as a Novel Prognostic Biomarker for Hepatocellular Carcinoma. Cancer Biomarkers Section A Dis Markers (2017) 18:209–14. doi: 10.3233/cbm-161671, PMID: 27983537 PMC13020593

[33] Lee J Gundara J Glover A Serpell J Sidhu S. MicroRNA Expression Profiles in the Management of Papillary Thyroid Cancer. Oncol (2014) 19:1141–7. doi: 10.1634/theoncologist.2014-0135, PMID: 25323484 PMC4221366

[34] Aragon Han P Weng C Khawaja H Nagarajan N Schneider E Umbricht C. MicroRNA Expression and Association With Clinicopathologic Features in Papillary Thyroid Cancer: A Systematic Review. Thyroid (2015) 25:1322–9. doi: 10.1089/thy.2015.0193, PMID: 26414548

[35] Cahill S Smyth P Denning K Flavin R Li J Potratz A. Effect of BRAFV600E Mutation on Transcription and Post-Transcriptional Regulation in a Papillary Thyroid Carcinoma Model. Mol Cancer (2007) 6:21. doi: 10.1186/1476-4598-6-21, PMID: 17355635 PMC1831483

[36] Jazdzewski K Boguslawska J Jendrzejewski J Liyanarachchi S Pachucki J Wardyn K. Thyroid Hormone Receptor Beta (THRB) Is a Major Target Gene for microRNAs Deregulated in Papillary Thyroid Carcinoma (PTC). J Clin Endocrinol Metab (2011) 96:E546–53. doi: 10.1210/jc.2010-1594, PMID: 21159845 PMC3047217

[37] Laukiene R Jakubkevicius V Ambrozaityte L Cimbalistiene L Utkus A. Dysregulation of microRNAs as the Risk Factor of Lymph Node Metastasis in Papillary Thyroid Carcinoma: Systematic Review. Endokrynol Pol (2021) 72:145–52. doi: 10.5603/EP.a2021.0010, PMID: 33970479

[38] Knyazeva M Korobkina E Karizky A Sorokin M Buzdin A Vorobyev S. Reciprocal Dysregulation of MiR-146b and MiR-451 Contributes in Malignant Phenotype of Follicular Thyroid Tumor. Int J Mol Sci (2020) 21:5950. doi: 10.3390/ijms21175950, PMID: 32824921 PMC7503510

[39] Pishkari S Paryan M Hashemi M Baldini E Mohammadi-Yeganeh S. The Role of microRNAs in Different Types of Thyroid Carcinoma: A Comprehensive Analysis to Find New miRNA Supplementary Therapies. J Endocrinol Invest (2018) 41:269–83. doi: 10.1007/s40618-017-0735-6, PMID: 28762013

[40] Shin J Shim H Hwang T Kim H Kang S Dho Y. Restoration of miR-29b Exerts Anti-Cancer Effects on Glioblastoma. Cancer Cell Int (2017) 17:104. doi: 10.1186/s12935-017-0476-9, PMID: 29176935 PMC5693545

[41] Zhao X Liu Y Li Z Zheng S Wang Z Li W. Linc00511 Acts as a Competing Endogenous RNA to Regulate VEGFA Expression Through Sponging hsa-miR-29b-3p in Pancreatic Ductal Adenocarcinoma. J Cell Mol Med (2018) 22:655–67. doi: 10.1111/jcmm.13351, PMID: 28984028 PMC5742682

[42] Wang H Ding Q Wang M Guo M Zhao Q. miR-29b inhibits the progression of multiple myeloma through downregulating FOXP1. Hematology (2019) 24:32–38. doi: 10.1080/10245332.2018.1502961, PMID: 30068241

[43] Medeiros M Ribeiro AO Lupi LA Romualdo GR Pinhal D Chuffa LGA. Mimicking the tumor microenvironment: Fibroblasts reduce miR-29b expression and increase the motility of ovarian cancer cells in a co-culture model. Biochem Biophys Res Commun (2019) 516:96–101. doi: 10.1016/j.bbrc.2019.06.001, PMID: 31200958

[44] Zhou Y Li K Dai T Wang H Hua Z Bian W. Long non-Coding RNA HCP5 Functions as a Sponge of miR-29b-3p and Promotes Cell Growth and Metastasis in Hepatocellular Carcinoma Through Upregulating DNMT3A. Aging (2021) 13:16267–86. doi: 10.18632/aging.203155, PMID: 34148029 PMC8266334

[45] Zhao W Huang Z Liu H Wang C. LncRNA GIHCG Promotes the Development of Esophageal Cancer by Modulating miR-29b-3p/ANO1 Axis. OncoTargets Ther (2020) 13:13387–400. doi: 10.2147/ott.S282348, PMID: 33408485 PMC7781470

[46] Gao D Niu Q Gong Y Guo Q Zhang S Wang Y. Y-Box Binding Protein 1 Regulates Angiogenesis in Bladder Cancer via miR-29b-3p-VEGFA Pathway. J Oncol (2021) 2021:9913015. doi: 10.1155/2021/9913015, PMID: 34306080 PMC8270724

[47] Huang G Li F Zhao X Ma Y Li Y Lin M. Functional and Biomimetic Materials for Engineering of the Three-Dimensional Cell Microenvironment. Chem Rev (2017) 117:12764–850. doi: 10.1021/acs.chemrev.7b00094, PMID: 28991456 PMC6494624

[48] Chen P Miao Y Zhang F Huang J Chen Y Fan Z. Nanoscale Microenvironment Engineering Based on Layer-by-Layer Self-Assembly to Regulate Hair Follicle Stem Cell Fate for Regenerative Medicine. Theranostics (2020) 10:11673–89. doi: 10.7150/thno.48723, PMID: 33052240 PMC7545990

[49] Marques-Magalhães Â Cruz T Costa Â Estêvão D Rios E Canão P. Decellularized Colorectal Cancer Matrices as Bioactive Scaffolds for Studying Tumor-Stroma Interactions. Cancers (2022) 14:359. doi: 10.3390/cancers14020359, PMID: 35053521 PMC8773780

[50] Kang J La Manna F Bonollo F Sampson N Alberts I Mingels C. Tumor Microenvironment Mechanisms and Bone Metastatic Disease Progression of Prostate Cancer. Cancer Lett (2022) 530:156–69. doi: 10.1016/j.canlet.2022.01.015, PMID: 35051532

[51] Ros M Sala M Saltel F. Linking Matrix Rigidity With EMT and Cancer Invasion. Dev Cell (2020) 54:293–95. doi: 10.1016/j.devcel.2020.06.032, PMID: 32781020

[52] Jiang J Wang K Chen Y Chen H Nice E Huang C. Redox Regulation in Tumor Cell Epithelial-Mesenchymal Transition: Molecular Basis and Therapeutic Strategy. Signal Transduct Target Ther (2017) 2:17036. doi: 10.1038/sigtrans.2017.36, PMID: 29263924 PMC5661624

[53] Nissen N Karsdal M Willumsen N. Collagens and Cancer Associated Fibroblasts in the Reactive Stroma and Its Relation to Cancer Biology. J Exp Clin Cancer Res CR (2019) 38:115. doi: 10.1186/s13046-019-1110-6, PMID: 30841909 PMC6404286

[54] Graf F Horn P Ho A Boutros M Maercker C. The Extracellular Matrix Proteins Type I Collagen, Type III Collagen, Fibronectin, and Laminin 421 Stimulate Migration of Cancer Cells. FASEB J (2021) 35:e21692. doi: 10.1096/fj.202002558RR, PMID: 34118087

[55] Peng D Ungewiss C Tong P Byers L Wang J Canales J. ZEB1 Induces LOXL2-Mediated Collagen Stabilization and Deposition in the Extracellular Matrix to Drive Lung Cancer Invasion and Metastasis. Oncogene (2017) 36:1925–38. doi: 10.1038/onc.2016.358, PMID: 27694892 PMC5378666

[56] Ray A Provenzano P. Aligned Forces: Origins and Mechanisms of Cancer Dissemination Guided by Extracellular Matrix Architecture. Curr Opin Cell Biol (2021) 72:63–71. doi: 10.1016/j.ceb.2021.05.004, PMID: 34186415 PMC8530881

[57] Kim M Ha S Wu M Zogg H Ronkon C Lee M. Extracellular Matrix Biomarkers in Colorectal Cancer. Int J Mol Sci (2021) 22:9185. doi: 10.3390/ijms22179185, PMID: 34502094 PMC8430714

[58] Vella V Nicolosi ML Cantafio P Massimino M Lappano R Vigneri P. DDR1 regulates thyroid cancer cell differentiation via IGF-2/IR-A autocrine signaling loop. Endocr Relat Cancer (2019) 26:197–214. doi: 10.1530/ERC-18-0310, PMID: 30121624

[59] Yoshida T Suganuma N Sato S Toda S Nakayama H Masudo K. Membrane Type 1 Matrix Metalloproteinase Regulates Anaplastic Thyroid Carcinoma Cell Growth and Invasion Into the Collagen Matrix. Biochem Biophys Res Commun (2020) 529:1195–200. doi: 10.1016/j.bbrc.2020.06.043, PMID: 32819585

[60] Parascandolo A Rappa F Cappello F Kim J Cantu D Chen H. Extracellular Superoxide Dismutase Expression in Papillary Thyroid Cancer Mesenchymal Stem/Stromal Cells Modulates Cancer Cell Growth and Migration. Sci Rep (2017) 7:41416. doi: 10.1038/srep41416, PMID: 28216675 PMC5316948

[61] Shi Y Duan Z Zhang X Zhang X Wang G Li F. Down-Regulation of the Let-7i Facilitates Gastric Cancer Invasion and Metastasis by Targeting COL1A1. Protein Cell (2019) 10:143–48. doi: 10.1007/s13238-018-0550-7, PMID: 29858755 PMC6340894

[62] Yu L Sui B Fan W Lei L Zhou L Yang L. Exosomes Derived From Osteogenic Tumor Activate Osteoclast Differentiation and Concurrently Inhibit Osteogenesis by Transferring COL1A1-Targeting miRNA-92a-1-5p. J Extracell Vesicles (2021) 10:e12056. doi: 10.1002/jev2.12056, PMID: 33489015 PMC7812369

[63] Rennhack J To B Swiatnicki M Dulak C Ogrodzinski M Zhang Y. Integrated Analyses of Murine Breast Cancer Models Reveal Critical Parallels With Human Disease. Nat Commun (2019) 10:3261. doi: 10.1038/s41467-019-11236-3, PMID: 31332182 PMC6646342

[64] Tian C Huang Y Clauser K Rickelt S Lau A Carr S. Suppression of Pancreatic Ductal Adenocarcinoma Growth and Metastasis by Fibrillar Collagens Produced Selectively by Tumor Cells. Nat Commun (2021) 12:2328. doi: 10.1038/s41467-021-22490-9, PMID: 33879793 PMC8058088

[65] Chakravarthy D Muñoz A Su A Hwang R Keppler B Chan D. Palmatine Suppresses Glutamine-Mediated Interaction Between Pancreatic Cancer and Stellate Cells Through Simultaneous Inhibition of Survivin and COL1A1. Cancer Lett (2018) 419:103–15. doi: 10.1016/j.canlet.2018.01.057, PMID: 29414301 PMC5858579

[66] Ma H Chang H Bamodu O Yadav V Huang T Wu A. Collagen 1a1 (COL1A1) Is a Reliable Biomarker and Putative Therapeutic Target for Hepatocellular Carcinogenesis and Metastasis. Cancers (2019) 11:786. doi: 10.3390/cancers11060786, PMID: 31181620 PMC6627889

[67] Geng Q Shen Z Li L Zhao J. COL1A1 Is a Prognostic Biomarker and Correlated With Immune Infiltrates in Lung Cancer. PeerJ (2021) 9:e11145. doi: 10.7717/peerj.11145, PMID: 33850663 PMC8018245

[68] Zhang Y Jing Y Wang Y Tang J Zhu X Jin W. NAT10 Promotes Gastric Cancer Metastasis *via* N4-Acetylated COL5A1. Signal Transduct Target Ther (2021) 6:173. doi: 10.1038/s41392-021-00489-4, PMID: 33941767 PMC8093205

[69] Liu W Wei H Gao Z Chen G Liu Y Gao X. COL5A1 May Contribute the Metastasis of Lung Adenocarcinoma. Gene (2018) 665:57–66. doi: 10.1016/j.gene.2018.04.066, PMID: 29702185

[70] Wu M Sun Q Mo C Pang J Hou J Pang L. Prospective Molecular Mechanism of COL5A1 in Breast Cancer Based on a Microarray, RNA Sequencing and Immunohistochemistry. Oncol Rep (2019) 42:151–75. doi: 10.3892/or.2019.7147, PMID: 31059074 PMC6549075

[71] Chen H Tseng Y Shu C Weng T Liou H Yen L. Differential Clinical Significance of COL5A1 and COL5A2 in Tongue Squamous Cell Carcinoma. J Oral Pathol Med (2019) 48:468–76. doi: 10.1111/jop.12861, PMID: 30972812

[72] Xue J Zhu S Qi F Zhu K Cao P Yang J. RUNX1/miR-582-5p Pathway Regulates the Tumor Progression in Clear Cell Renal Cell Carcinoma by Targeting Col5a1. Front Oncol (2021) 11:610992. doi: 10.3389/fonc.2021.610992, PMID: 33937021 PMC8079757

