# Correction: MiR-29b-3p inhibits migration and invasion of papillary thyroid carcinoma by down-regulating COL1A1 and COL5A1

**DOI:** 10.3389/fonc.2025.1657780

**Published:** 2025-10-03

**Authors:** Congjun Wang, Ye Wang, Zhao Fu, Weijia Huang, Zhu Yu, Jiancheng Wang, Kaitian Zheng, Siwen Zhang, Shen Li, Junqiang Chen

**Affiliations:** ^1^ Department of Gastrointestinal Gland Surgery, The First Affiliated Hospital of Guangxi Medical University, Nanning, China; ^2^ Clinical Research Lab, Guangxi Key Laboratory of Enhanced Recovery After Surgery for Gastrointestinal Cancer, Nanning, China; ^3^ Department of Gastrointestinal, Hernia and Enterofistula Surgery, The People’s Hospital of Guangxi Zhuang Autonomous Region, Nanning, China

**Keywords:** microRNAs, miR-29b-3p, papillary thyroid carcinoma, downregulation, COL1A1, COL5A1, gene expression

In the published article, there was an error in **Figure 2** as published. In **Figure 2E**, the EDU experimental result images for the B-CPAP cell line NC inhibitor treatment group were misplaced. The corrected **Figure 2** and its caption appear below.

**Figure 2:**
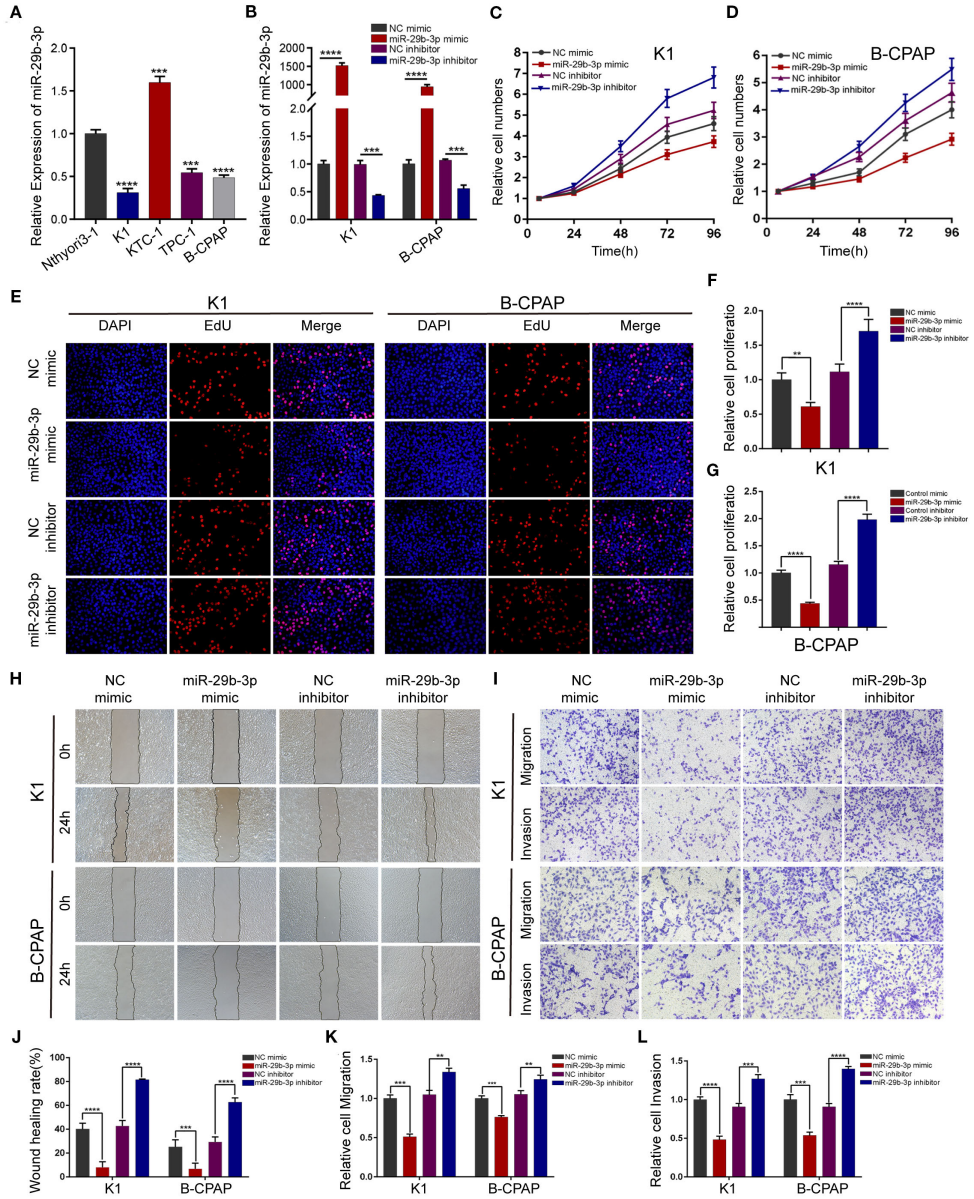
miR-29b-3p inhibits PTC cell migration and invasion in vitro. **(A)** Expression of miR-29b-3p was verified by qRT-PCR in PTC cell lines (K1, KTC-1, TPC-1, and B-CPAP) and normal thyroid cell lines (Nthyori3–1); two lowly expressing (K1 and B-CPAP) cell lines were selected for further study. **(B)** The knockdown and overexpression efficiencies of miR-29b-3p was verified by qRT-PCR after transfection with miR-29b-3p mimic/inhibitor or NC mimic/inhibitor. Cell proliferation ability of PTC cells was determined by CCK-8 assays **(C, D)** and EdU assays **(E–G)** after miR-29b-3p overexpression or knockdown. The effects of miR-29b-3p overexpression and knockdown in PTC cells on cell migration and invasion were analyzed by wound healing assays **(H)** and Transwell assays **(I)**. **(J, L)** Results of the Transwell assays and wound healing assays. In the figure, **p < 0.01, ***p < 0.001,****p < 0.0001.

In the published article, there was an error in **Figure 4** as published. In **Figure 4N**, image arrangement errors occurred for the K1 cell line miR-29b-3p Mimic treatment group and the B-CPAP cell line NC Mimic, miR-29b-3p Mimic, and miR-29b-3p Mimic + OE COL5A1 treatment groups. The corrected **Figure 4** and its caption appear below.

**Figure 4:**
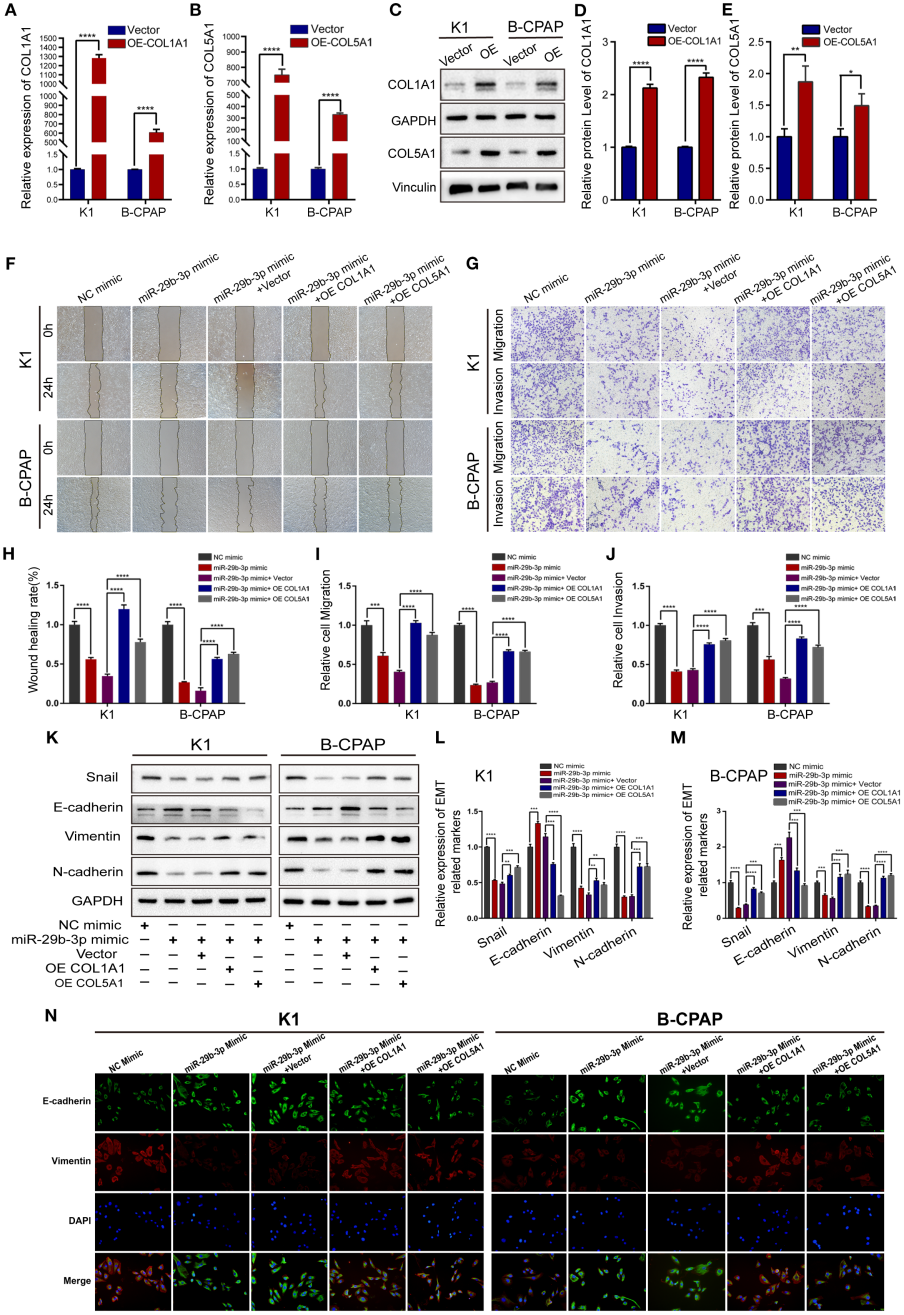
Overexpression of COL1A1 or COL5A1 partially blocks the suppressive effects of miR-29b-3p on invasion in PTC cells. **(A, B)** qRT-PCR and **(C)** Western blot were performed to assess the transfection efficiency of COL1A1 and COL5A1 overexpression plasmids. **(D, E)** Quantification of COL1A1 and COL5A1 levels in Western blot normalized over GAPDH and Vinculin. The wound healing assays **(F)** and Transwell assays **(G)** demonstrated that the overexpression of COL1A1 and COL5A1 could efficiently alleviate the miR-29b-3p mimic-induced inhibition of the migration and invasion activities of K1 and B-CPAP cells. **(H–J)** Quantified results of the wound-healing assays and Transwell assays. Inhibition of miR-29b-3p mimic-induced EMT by overexpressed COL1A1 or COL5A1; the EMT-related proteins were investigated by **(K)** Western blot and **(N)** immunofluorescence. **(L, M)** The EMT-related proteins expression for each group was analyzed by Image J software. In the figure, *p < 0.05, **p < 0.01, ***p < 0.001, ****p < 0.0001.

In the published article, there was an error in **Figure 5** as published. In **Figure 5L**, the immunofluorescence experimental result images for the B-CPAP cell line si-COL1A1–2 treatment group were misplaced during assembly. The corrected **Figure 5** and its caption appear below.

**Figure 5:**
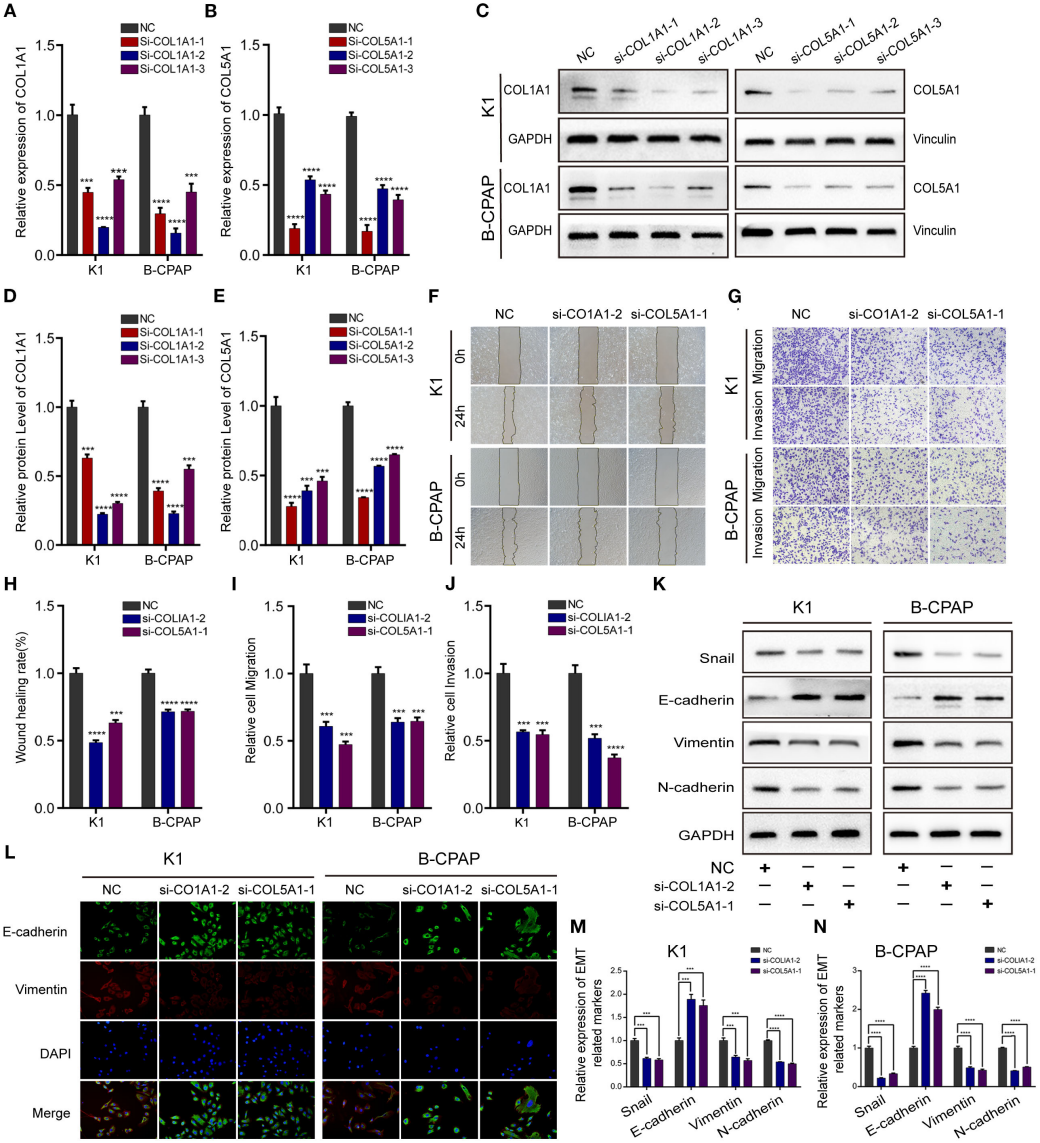
Down-regulating COL1A1 or COL5A1 expression can inhibit migration, invasion, and EMT in PTC cells. The knockdown efficiency of COL1A1 and COL5A1 were analyzed by **(A, B)** qRT-PCR and **(C–E)** and Western blotting. Additionally, **(F)** wound healing and **(G)** Transwell assays were conducted to evaluate PTC cells migration and invasion, which proved that both si-COL1A1–2 and si-COL5A1–1 significantly impaired migration and invasion ability in K1 and B-CPAP cells. **(H–J)** The statistical results of wound healing assays and Transwell assays. **(K)** Western blot and **(L)** immunofluorescence analyses were performed to determine the expression of EMT-related proteins detection in PTC cells transfected with si-COL1A1–2 and si-COL5A1–1. (M, N) Western blotting assay showing the protein expression levels of EMT-related in PTC cells. In the figure, ***p < 0.001, ****p < 0.0001.

The original version of this article has been updated.

