# Correction: MiR‑29b‑3p Inhibits Migration and Invasion of Papillary Thyroid Carcinoma by Downregulating COL1A1 and COL5A1

**DOI:** 10.3389/fonc.2026.1995652

**Published:** 2026-10-06

**Authors:** Congjun Wang, Ye Wang, Zhao Fu, Weijia Huang, Zhu Yu, Jiancheng Wang, Kaitian Zheng, Siwen Zhang, Shen Li, Junqiang Chen

**Affiliations:** 1 Department of Gastrointestinal Gland Surgery, The First Affiliated Hospital of Guangxi Medical University, Nanning, China; 2 Clinical Research Lab, Guangxi Key Laboratory of Enhanced Recovery After Surgery for Gastrointestinal Cancer, Nanning, China; 3 Department of Gastrointestinal, Hernia and Enterofistula Surgery, The People’s Hospital of Guangxi Zhuang Autonomous Region, Nanning, China

**Keywords:** microRNAs, miR-29b-3p, papillary thyroid carcinoma, downregulation, COL1A1, COL5A1, gene expression

In the published article, the **Reference** for “In turn, miR-29b-3p is a major regulator of the collagen family, which has been studied in a plurality of basic studies in the context of a range of diseases.” was erroneously written as “20. Tao R, Fan X, Yu H, Ai G, Zhang H, Kong H, et al. MicroRNA-29b-3p Prevents Schistosoma Japonicum-Induced Liver Fibrosis by Targeting COL1A1 and COL3A1. J Cell Biochem (2018) 119:3199–209. doi: 10.1002/jcb.26475”. It should be “20. Liang JN, Zou X, Fang XH, Xu JD, Xiao Z, Zhu JN, et al. The Smad3-miR-29b/miR-29c axis mediates the protective effect of macrophage migration inhibitory factor against cardiac fibrosis. Biochim Biophys Acta Mol Basis Dis (2019) 1865:2441-2450. doi:10.1016/j.bbadis.2019.06.004”.

The **Reference** for “Subsequent studies supported that miR-29b-3p could suppress the growth of multiple myeloma and ovarian cancer cells.” was erroneously written as “42. Liu D, Wang J, Liu M. Long Noncoding RNA TUG1 Promotes Proliferation and Inhibits Apoptosis in Multiple Myeloma by Inhibiting miR-29b-3p. Biosci Rep (2019) 39:2489. doi: 10.1042/bsr20182489”. It should be “42. Wang H, Ding Q, Wang M, Guo M, Zhao Q. miR-29b inhibits the progression of multiple myeloma through downregulating FOXP1. Hematology (2019) 24:32-38. doi:10.1080/10245332.2018.1502961”.

The **Reference** for “Subsequent studies supported that miR-29b-3p could suppress the growth of multiple myeloma and ovarian cancer cells.” was erroneously written as “43. Yang X, Xin N, Qu H, Wei L, Han Z. Long Noncoding RNA TUG1 Facilitates Cell Ovarian Cancer Progression Through Targeting MiR-29b-3p/MDM2 Axis. Anat Rec (Hoboken NJ 2007) (2020) 303:3024–34. doi: 10.1002/ar.24367”. It should be “43. Medeiros M, Ribeiro AO, Lupi LA, Romualdo GR, Pinhal D, Chuffa LGA, et al. Mimicking the tumor microenvironment: Fibroblasts reduce miR-29b expression and increase the motility of ovarian cancer cells in a co-culture model. Biochem Biophys Res Commun (2019) 516:96-101. doi: 10.1016/j.bbrc.2019.06.001”.

The **Reference** for “The progression of bladder cancer by inhibiting the proliferative, migratory, and invasive abilities of oncocytes.” was erroneously written as “46. Zhao C, Li Y, Hu X, Wang R, He W, Wang L, et al. LncRNA HCP5 Promotes Cell Invasion and Migration by Sponging miR-29b-3p in Human Bladder Cancer. OncoTargets Ther (2020) 13:11827–38. doi: 10.2147/ott.S249770”. It should be “46.Gao D, Niu Q, Gong Y, Guo Q, Zhang S, Wang Y, et al. Y-Box Binding Protein 1 Regulates Angiogenesis in Bladder Cancer via miR-29b-3p-VEGFA Pathway. J Oncol (2021) 2021:9913015. doi: 10.1155/2021/9913015”.

The **Reference** for “Moreover, collagens have also been demonstrated to be involved in the initiation and progression of thyroid cancer.” was erroneously written as “58. Geng H, Guo M, XuW, Zang X,Wu T, Teng F, et al. SHCBP1 Promotes Papillary Thyroid Carcinoma Carcinogenesis and Progression Through Promoting Formation of Integrin and Collagen and Maintaining Cell Stemness. Front Endocrinol (2020) 11:613879. doi: 10.3389/fendo.2020.613879”. It should be “58. Vella V, Nicolosi ML, Cantafio P, Massimino M, Lappano R, Vigneri P, et al. DDR1 regulates thyroid cancer cell differentiation via IGF-2/IR-A autocrine signaling loop. Endocr Relat Cancer (2019) 26:197-214. doi:10.1530/ERC-18-0310”.

The original version of this article has been updated.

